# Evolutionary diversification of methanotrophic ANME-1 archaea and their expansive virome

**DOI:** 10.1038/s41564-022-01297-4

**Published:** 2023-01-19

**Authors:** Rafael Laso-Pérez, Fabai Wu, Antoine Crémière, Daan R. Speth, John S. Magyar, Kehan Zhao, Mart Krupovic, Victoria J. Orphan

**Affiliations:** 1 MARUM, Center for Marine Environmental Science, and Department of Geosciences, University of Bremen, Bremen, Germany; 2 ZJU-Hangzhou Global Scientific and Technological Innovation Center, Hangzhou, China; 3 Ocean College, Zhejiang University, Zhoushan, China; 4 Donghai Laboratory, Zhoushan, China; 5 Division of Geological and Planetary Sciences, California Institute of Technology, Pasadena, CA USA; 6 Division of Biology and Biological Engineering, California Institute of Technology, Pasadena, CA USA; 7 Institut Pasteur, Université Paris Cité, CNRS UMR6047, Archaeal Virology Unit, Paris, France; 8 Present Address: Systems Biology Department, Centro Nacional de Biotecnología (CNB-CSIC), Madrid, Spain; 9 Present Address: Max-Planck Institute for Marine Microbiology, Bremen, Germany

**Keywords:** Microbial ecology, Virology, Biogeochemistry

## Abstract

‘*Candidatus* Methanophagales’ (ANME-1) is an order-level clade of archaea responsible for anaerobic methane oxidation in deep-sea sediments. The diversity, ecology and evolution of ANME-1 remain poorly understood. In this study, we use metagenomics on deep-sea hydrothermal samples to expand ANME-1 diversity and uncover the effect of virus–host dynamics. Phylogenetic analyses reveal a deep-branching, thermophilic family, ‘*Candidatus* Methanospirareceae’, closely related to short-chain alkane oxidizers. Global phylogeny and near-complete genomes show that hydrogen metabolism within ANME-1 is an ancient trait that was vertically inherited but differentially lost during lineage diversification. Metagenomics also uncovered 16 undescribed virus families so far exclusively targeting ANME-1 archaea, showing unique structural and replicative signatures. The expansive ANME-1 virome contains a metabolic gene repertoire that can influence host ecology and evolution through virus-mediated gene displacement. Our results suggest an evolutionary continuum between anaerobic methane and short-chain alkane oxidizers and underscore the effects of viruses on the dynamics and evolution of methane-driven ecosystems.

## Abstract (web summary)

ANME-1 archaea are important because of their ability to metabolize methane through anaerobic oxidation. Here the authors use metagenomics on hydrothermal samples from the Gulf of California to characterize a family of ANME-1 and its virome.

## Main

Anaerobic methanotrophic archaea (ANME) is a polyphyletic group of archaeal lineages that have independently evolved the ability of anaerobic oxidation of methane (AOM), a process that is estimated to remove more than 80% of the methane produced globally in deep-sea sediments^1^ by reversing the methanogenesis pathway^2^. Whereas the ANME-2 and ANME-3 lineages share common ancestors with the present-day methanogens of the *Methanosarcinales* order, ANME-1 archaea form their own order ‘*Candidatus* Methanophagales’, which is sister to the non-methane alkane degraders ‘*Candidatus* Syntrophoarchaeales’ and ‘*Candidatus* Alkanophagales’^3^. ANME-1 can grow beyond the cold and temperate deep-sea habitats that they often share with other ANMEs, uniquely thriving at higher temperatures within hydrothermal environments^2,4,5^. In marine sediments, ANMEs mostly form syntrophic associations with sulfate-reducing bacteria^6^ via direct interspecies electron transfer^7,8^. However, some ANME-1 have been observed as single cells or as monospecific consortia without partner bacteria^5,9–11^, and have been proposed to perform hydrogenotrophic methanogenesis^10–12^, although physiological experiments have thus far failed to support this hypothesis^13,14^. Overall, it remains largely unclear what factors have contributed to the physiological and ecological diversification of ANME-1 from their short-chain alkane relatives and other ANME lineages.

Despite the dominance of ANME archaea in many methane-rich ecosystems, viruses targeting ANME lineages are largely unexplored^15–17^. By exploiting and spilling host cellular resources through their replication and lytic cycles, viruses play a major role in the ecological dynamics and nutrient cycling in diverse microbial systems^18^. In deep-sea ecosystems, viral lysis has been estimated to cause annual archaeal mortality that releases up to around 0.3–0.5 gigatons of carbon globally^19^. Characterizing the distributions and functions of viruses of ANMEs is thus one of the most important tasks for quantitatively linking ANME physiology to the elemental and energy flows in deep-sea methane-driven ecosystems, and understanding the drivers of ANME evolution.

## Results

### A unique ANME-1 clade from hydrothermal vents

**Fig. 1:**
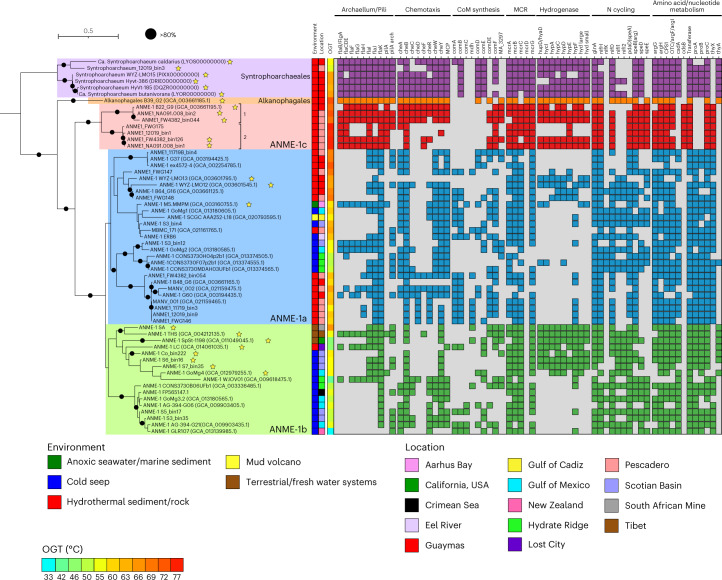
Phylogenomic tree and lineage differentiation of the ANME-1 order. In bold, genomes retrieved from the South Pescadero Basin. The colour bars indicate from left to right: environment, location, predicted OGT (in °C) and a genomic comparison of some metabolic features (main text and Supplementary Information). Numbers after the ANME-1c names indicate the two species of ANME-1c and the stars after the names denote MAGs containing at least the large subunit of a NiFe hydrogenase. Black circles indicate bootstrap support values over 80%. The scale bar represents the number of nucleotide substitutions per site. CoM, Coenzyme M.

**Extended Data Fig. 1:**
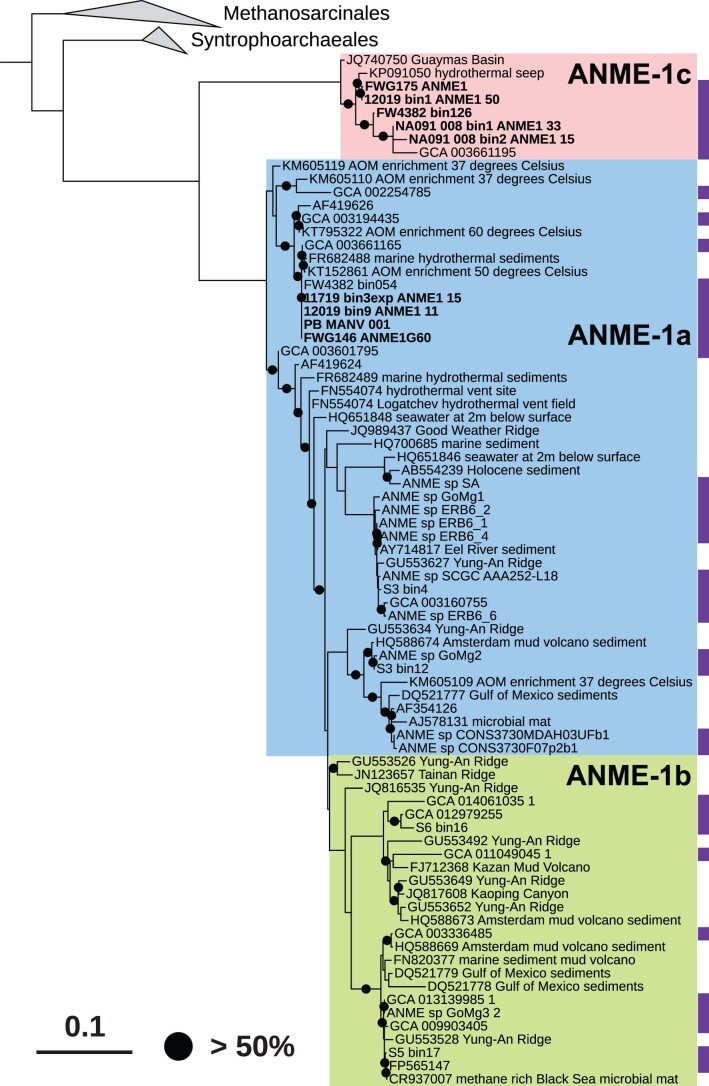
16S rRNA gene phylogeny for the ANME-1 clade (*Methanophagales*). Color shading highlights the three main groups of ANME-1 archaea. The purple bars note 16S rRNA gene sequences retrieved from MAGs shown in Fig. 1. Sequences retrieved from Pescadero MAGs are in bold. Bootstrap values over 50% are indicated with a black circle. Scale bar indicates the number of nucleotide substitutions per site.

In this study, we recovered 13 metagenome-assembled genomes (MAGs) of ANME-1 in native and laboratory-incubated mineral samples from the Southern Pescadero Basin hydrothermal vent system^20^ in the Gulf of California, Mexico (Supplementary Tables 1 and 2). These samples not only expanded the known diversity within the ANME-1a clade, particularly the ANME-1 G60 group, but also contained five MAGs and one 1.6 Mb circular genome scaffold of a previously uncharacterized deep-branching clade phylogenetically positioned at the base of the ANME-1 order (Fig. 1, Extended Data Fig. 1 and Supplementary Tables 2 and 3). We name this family-level clade ‘*Candidatus* Methanospirareceae’, or ANME-1c. Given its basal position, it is the phylogenetically closest ANME-1 to the sister orders of non-methane alkane degraders Alkanophagales and Syntrophoarchaeales^21^.

**Fig. 2:**
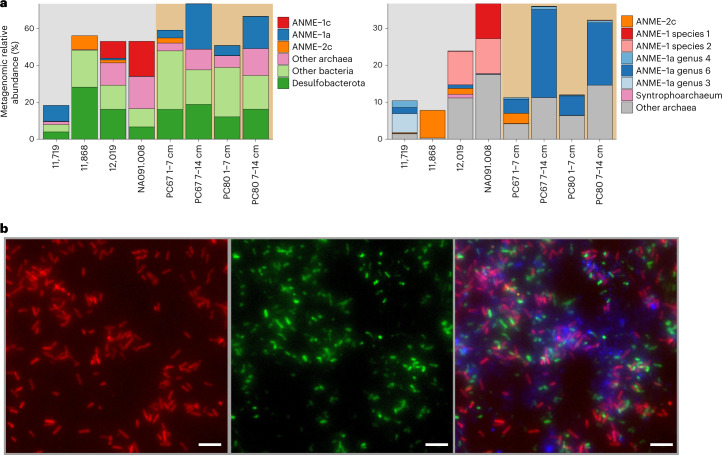
Distribution and morphology of ANME-1 in South Pescadero sediment and rock samples. **a**, Relative abundance of MAGs from ANME and other bacteria and archaeal lineages (left). Genomic abundance for archaea at the species level is on the right, highlighting a variation ANME-1 lineages in rocks and sediments (right). Colour background indicates rock (grey) or sediment (brown) samples. The total abundance does not reach 100% because unmapped reads are not included. Note the different scales of the *y* axes between panels. **b**, Fluorescence in situ hybridization of ANME-1 cells recovered from rock sample NA091.008 (*n* = 2). Cells targeted by the general ANME-1-350 probe are shown in red (left). Cells targeted by the general bacterial probe 338 are in green (middle). A composite overlay showing bacteria (green), ANME-1 cells (red) and DAPI staining of all microbial cells in blue (right). Scale bar, 5 µm.

Our ANME-1c MAGs represent two different genera, ‘*Candidatus* Methanoxibalbensis’ and ‘*Candidatus* Methanospirare’ within the same family with an average nucleotide identity of 76%, represented by species ‘*Candidatus* Methanoxibalbensis ujae’ (species 1) and ‘*Candidatus* Methanospirare jalkutatii’ (species 2, Methods). Based on genome coverage, these two ANME-1 species were the most abundant organisms in rock samples 12,019 and NA091.008, whereas they were hardly detected in rocks 11,868 and 11,719 and in hydrothermal sediments (Fig. 2a).

**Extended Data Fig. 2:**
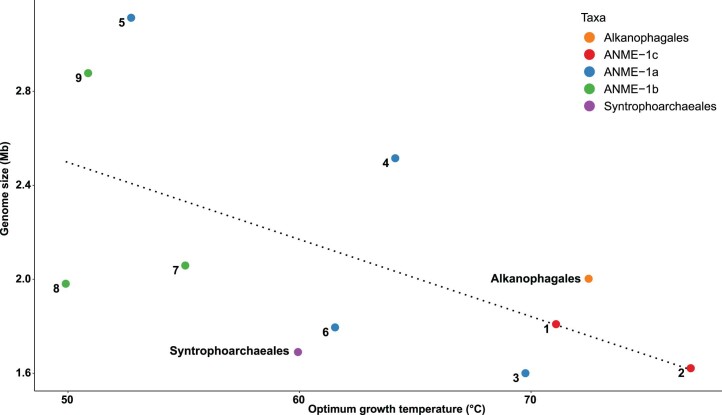
Correlation between estimated genome size (in Mb and after calculation considering contamination and completeness see Material and Methods) and the predicted optimum growth temperature (°C). Each point and number represents the average values for one ANME genera/species (see Supplementary Table 2), except in the case of *Syntrophoarchaeales* and *Alkanophagales* where the point represent the average values for the whole clade. Color indicates the corresponding taxonomy. Dotted line indicates the regression model (R^2^ = 0.3858).

So far, all ANME-1c MAGs and 16S rRNA gene sequences from the National Center for Biotechnology Information (NCBI; https://www.ncbi.nlm.nih.gov/) and SILVA (https://www.arb-silva.de/) databases have originated from hydrothermal environments, specifically the sediments of Guaymas^11,22^ and Southern Pescadero basins^23^. These hydrothermal habitats are 400 km apart along the same fault system in the Gulf of California and exhibit 20% species-level overlap in the microbial community^23^. This distribution suggests a strong thermophilic physiological specialization of ANME-1c to hydrothermal environments. Indeed, genome-based prediction^24^ suggested a high theoretical optimal growth temperature (OGT; Supplementary Table 4 and Extended Data Fig. 2) for both ANME-1c species (>70 °C) that was higher than the average predicted OGT for both ANME-1a (62 °C) and ANME-1b (52 °C). Such high temperature adaptation by ANME-1c could be related to their reduced estimated genome size (‘*Candidatus* Methanoxibalbensis ujae’: 1.81 Mb; ‘*Candidatus* Methanospirare jalkutatii’: 1.62 Mb) as previously observed in other thermophilic bacteria and archaea^25^.

Using fluorescence in situ hybridization with an ANME-1-targeted 16S rRNA probe, we detected ANME-1 cells in rock NA091.008 (Fig. 2b), where ANME-1c were the dominant lineage according to genome coverage (Fig. 2a). These putative ANME-1c cells exhibit the typical cylindrical shape previously reported for other ANME-1 populations^6^ and were loosely associated with bacterial cells in an extracellular polymeric substances matrix, or found as single cells.

### Physiological differentiation of diverse ANME-1 archaea

The deep-branching position of ANME-1c led us to examine the genomic patterns of emergence and differentiation of ANME-1 from the sister orders Alkanophagales and Syntrophoarchaeales. Like all ANME-1, ANME-1c encode a complete reverse methanogenesis pathway including a single operon for the methyl coenzyme M reductase enzyme (MCR), responsible for the activation of methane, and the replacement of F_420_-dependent methylene-H_4_MPT reductase by 5,10-methylenetetrahydrofolate reductase characteristic for ANME-1^2,8^. Similar to other ANME clades, ANME-1c encodes several multiheme cytochromes, which likely mediate the transfer of electrons during syntrophic AOM to sulfate-reducing bacteria^2,7,8^.

**Extended Data Fig. 3:**
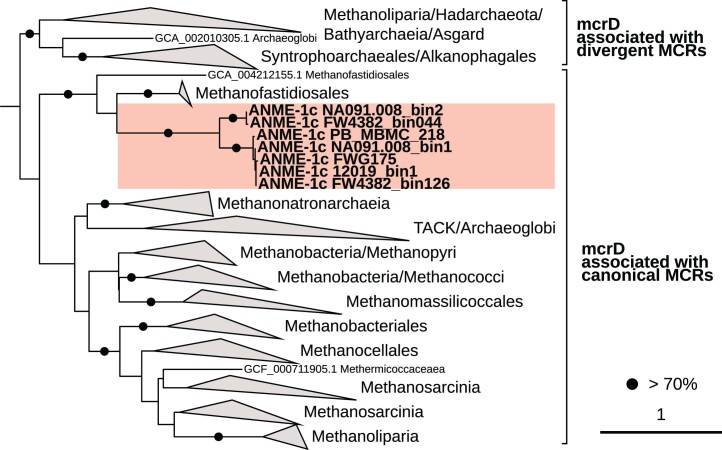
Phylogenetic tree of McrD genes from archaea, including the McrD in ANME-1 genomes (only found in ANME-1c). Black circles indicate bootstrap support values over 70%. Scale bar represents the number of amino acid substitutions per site.

Notably, ANME-1c exhibit distinct features compared to the ANME-1a and ANME-1b in the operon encoding the MCR enzyme. This enzyme consists of six subunits with the structure α_2_β_2_ϒ_2_ and the unique nickel-containing cofactor coenzyme F_430_ (ref. 26). In the maturation of this cofactor, McrC and McrD, two additional proteins encoded by the MCR operon in methanogens, are involved^27,28^. Although *mcrD* is not present in ANME-1a and ANME-1b, both genes are present in ANME-1c, where *mcrD* forms an operon with *mcrABG* (Fig. 1). Previous analysis suggested that ANME-1 acquired the *mcr* genes from distant H_2_-dependent methylotrophic methanogens of the class Methanofastidiosa^2^, whereas they lost the divergent MCRs present in Syntrophoarchaeales and Alkanophagales, which seem to use larger alkanes. Likewise, we found that the ANME-1c McrD is closely related to the McrD of Methanofastidiosa but only distantly related to the McrD of Syntrophoarchaeales and Alkanophagales that form a different cluster (Extended Data Fig. 3). These results suggest that during the emergence of ANME-1, a full operon of methane-cycling *mcr* (including *mcrCD*) was acquired by horizontal gene transfer from a Methanofastidiosa-related methylotrophic methanogen, and *mcrD* was later lost in both ANME-1a and ANME-1b clades. The ANME-1c also exhibit several additional genomic features that are distinct, highlighted in Fig. 1 and described in Supplementary Information and Supplementary Table 5.

### Shared origin and differential loss of hydrogenases

**Fig. 3:**
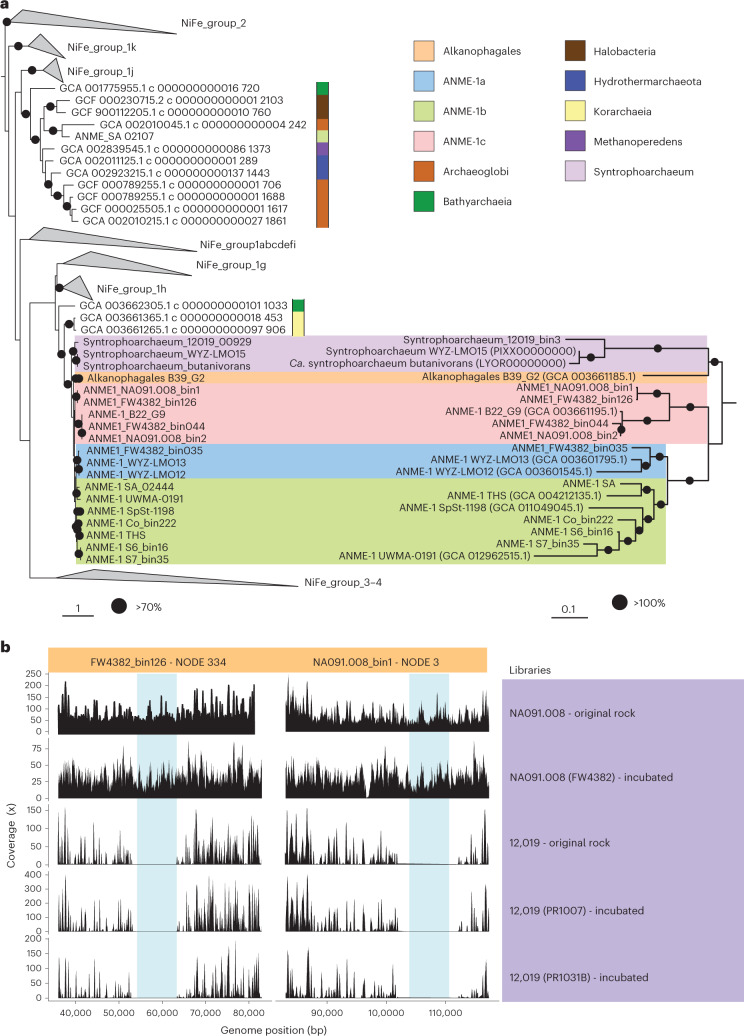
Vertical inheritance and differential loss of hydrogenases across ANME-1. **a**, Phylogenetic tree of the large subunit of the NiFe hydrogenase present in ANME-1 genomes (left) and the corresponding phylogenomic tree of those genomes (right). A few NiFe hydrogenases of ANME-1 genomes were also affiliated with NiFe Group 3 and 4 (not shown, see Extended Data Fig. 5). Colour bars/backgrounds indicate the phylogenetic affiliation of hydrogenases of interest. Black circles indicate bootstrap support values over 70% (left) and equal to 100% (right). Scale bars represent the number of amino acid substitutions per site. **b**, Read coverage distribution of the hydrogenase operon of ANME-1c genomes FW4382_bin126 and NA091.008_bin1. Metagenomic read libraries are indicated on the right. The blue shade indicates where the hydrogenase operon is located within the corresponding contig.

**Extended Data Fig. 4:**
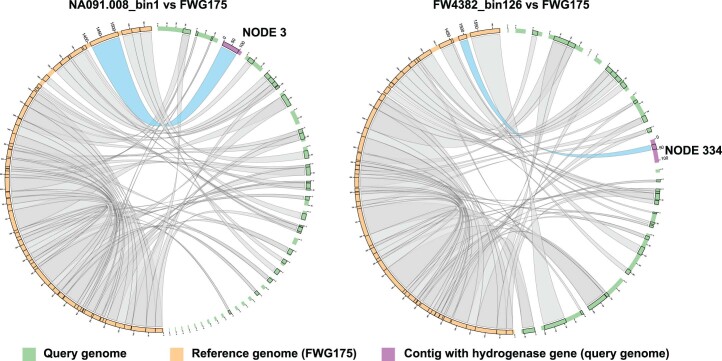
Circos plot comparing homologous regions of the ANME-1c genomes, NA091.008_bin1 and FW4382_bin126 (both with hydrogenase operons) to the predicted completed genome FWG175 that was assembled as a single contiguous scaffold and belongs to the same species. Contigs corresponding to the query genomes (NA091.008, FW4382_bin126) are marked in green and the genome scaffold of FWG175 are in orange. The contig containing the hydrogenase operon is shown in purple and the corresponding homology sections between the reference and query genomes are highlighted in blue. The region between these homology sections corresponds to the hydrogenase operon that was not detected in genome FWG175.

Hydrogen was proposed as one of the first candidate intermediates in syntrophic AOM, but fell out of favour after several genomic studies showed that the majority of ANME genomes do not encode hydrogenases. However, recent studies have reported NiFe-hydrogenases in subclades of larger ANME groups, including an ANME-1b subclade ‘*Candidatus* Methanoalium’ and from select ANME-1a genomes (Fig. 1)^2,29^. Interestingly, the genomes of the sister orders Syntrophoarchaeales and Alkanophagales encode a NiFe hydrogenase (Fig. 1), but physiological experiments did not support a role of this hydrogenase in syntrophic alkane oxidation^21^. Our expanded phylogenomic analysis of ANME-1 confirm that genomes associated with three distinct subclades of the ANME-1a, ANME-1b and now ANME-1c each encode a NiFe hydrogenase operon (Fig. 1). Phylogenetic analysis of the large subunit of these hydrogenases revealed a monophyletic group of ANME-1-affiliated hydrogenases clustering with those of Syntrophoarchaeales and Alkanophagales (Fig. 3a and Supplementary Table 6). Hence, the occurrence of hydrogenases appears to be an ancient trait of the class *Syntrophoarchaeia* that was vertically inherited by the common ancestor of ANME-1 and later differentially lost during ANME-1 clade diversification. Strikingly, the occurrence of hydrogenase has an apparent mosaic distribution among MAGs even within the hydrogenase-containing clades. For instance, within ANME-1c, only two out of five MAGs of ‘*Candidatus* Methanospirare jalkutatii’ (FW4382_bin126 and NA091.008_bin1) encode hydrogenases, whereas the complete ‘*Candidatus* Methanospirare jalkutatii’ MAG FWG175, assembled into a single scaffold, does not contain them. To verify that this distribution is caused by the intraspecies variation rather than incomplete genome assembly, we conducted independent metagenomic analyses that confirmed the differential presence of hydrogenase genes within ANME strains of different rock samples (Fig. 3b, Extended Data Fig. 4 and Supplementary Information). Hydrogenases thus appear to be a part of the pangenomic repertoire of certain ANME-1 subclades and species, likely preserved in the ANME-1 pangenome as an environmental adaptation rather than as an absolute requirement for the methanotrophic core metabolism.

**Extended Data Fig. 5:**
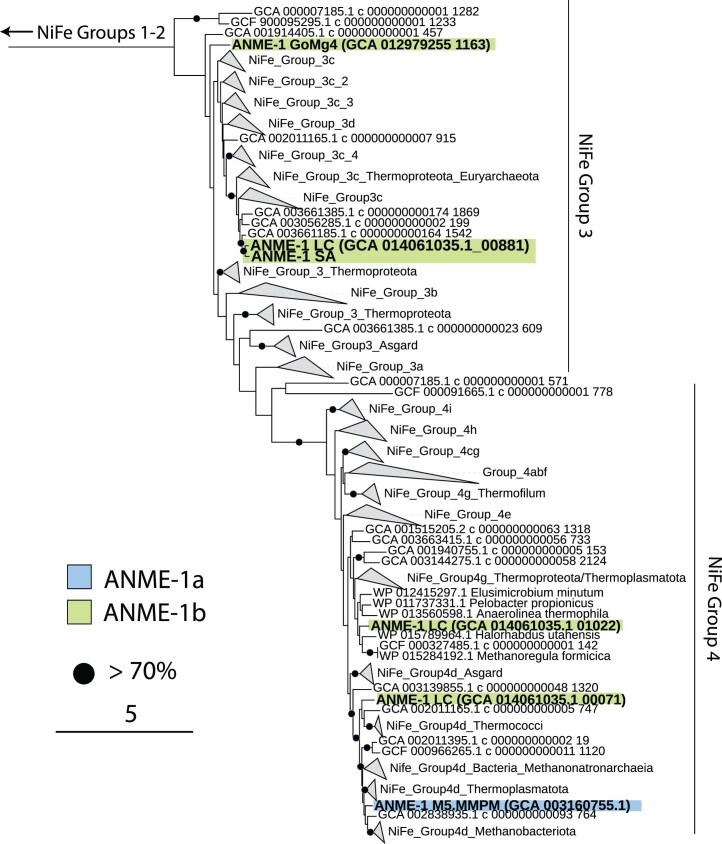
Phylogenetic tree of the large subunit of the NiFe hydrogenase present in ANME-1 genomes associated with NiFe Groups 3 and 4. The green and blue shading indicates the taxonomic identity of the ANME-1 MAG containing the corresponding hydrogenase. Black circles indicate bootstrap support values over 70%. The scale bar represents the number of amino acid substitutions per site.

The potential role of these hydrogenases in ANME-1 is still unclear. Their phylogenetic position, next to hydrogenotrophic enzymes^30^ of the NiFe groups 1g and 1h (Fig. 3a; only a few affiliated to NiFe Group 3 and 4, Extended Data Fig. 5), suggest a possible involvement in hydrogenotrophic methanogenesis, as previously proposed based on biochemical^31^, environmental^11,12^, isotopic^10^ and metagenomic data^29^, although enrichment cultivation attempts with hydrogen have been unsuccessful^13,14^. Recently, the genomic analysis of the hydrogenase-encoding ANME-1b group ‘*Candidatus* Methanoalium’ showed the presence of distinct electron-cycling features (Rnf complex, cytochrome b) and the absence of multiheme cytochromes suggesting a methanogenic metabolism for this group^2^. By contrast, ANME-1c encodes multiheme cytochromes and lacks these electron-cycling features. Hence, the physiological utility of hydrogenases may vary between lineages. Whereas hydrogen is likely not feasible as the sole intermediate for syntrophic AOM^7,13^, it could be produced by ANME-1c as an additional intermediate, as proposed in a mixed model involving direct electron transfer and metabolite exchange^2,32^.

### CRISPR-based discovery of an expansive ANME-1 mobilome

**Fig. 4:**
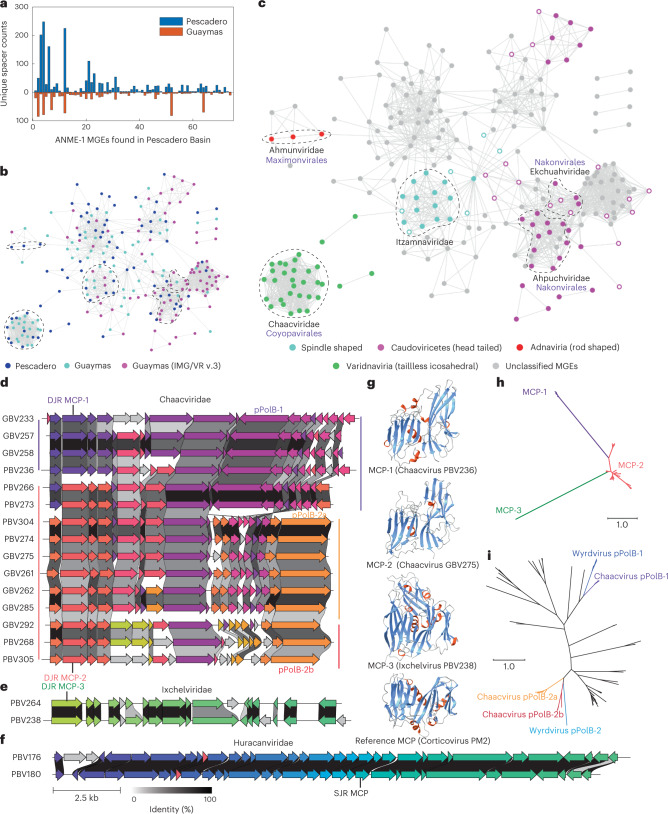
Expansive ANME-1 mobilome includes 16 undescribed viral families and structurally predicted MCPs. **a**, Histograms showing the number of CRISPR spacers from South Pescadero and Guaymas basin metagenomes matching the South Pescadero ANME-1 MGEs. **b**, Gene sharing network of diverse ANME-1 MGEs of different origins. **c**, ANME-1 MGEs, exhibited in the same network as **b**, are found to encompass major archaeal virus diversity and non-viral elements. Solid or open circles indicate viral assemblies with/without identifiable MCPs. In (**b**) and (**c**), dashed lines encircle five proposed viral families containing complete genome representatives. The proposed names of viral families (black) and orders (purple) are indicated in (**c**). **d**–**f**, Gene synteny of three proposed families of tailless icosahedral viruses targeting ANME-1. Different colours indicate 83 different protein groups. Grey shading denotes singletons. The scale bar and precent identity shading are indicated in (**f**). **g**, Alphafold2-predicted structures of DJR MCPs in ANME-1 viruses shown in (**d**) and (**e**). Blue indicates **β** barrels, and red **α** helices. **h**, Maximum-likelihood analysis of proposed MCP families indicates their long evolutionary distances. **i**, Maximum-likelihood analysis of PolB found in different clades of the tailless Chaacviruses targeting ANME-1 archaea are related to two clades of spindle-shaped Wyrdviruses targeting Asgard archaea. SJR, single jelly-roll.

**Extended Data Fig. 6:**
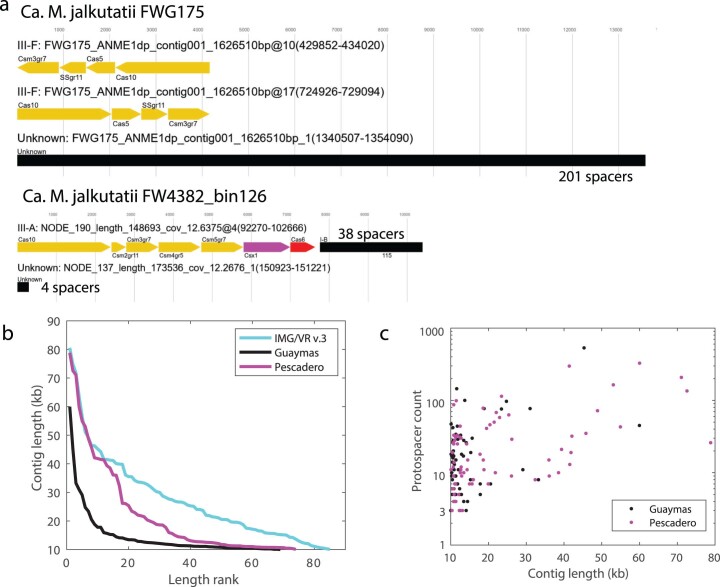
Features of ANME-1 CRISPR/Cas and spacer-mobilome mapping. (**a**) CRISPR/Cas features in the two most contiguous ANME-1c MAGs characterized using CCtyper. Black bars indicate CRISPR arrays. (**b**) Contig lengths of all ANME-1 mobile genetic elements (MGEs) found in this study. Note that contig length does not necessarily indicate completeness as IMG/VR v.3 is more enriched with head-tailed viruses (with genomes sized up 80 kb) whereas the contigs obtained directly from Pescadero/Guaymas basin metagenomic assemblies contain many tailless icosahedral viruses whose genomes are sized around 10 kb. (**c**) Distribution of protospacers within the ANME-1 mobile elements found in S. Pescadero and Guaymas basins.

**Extended Data Fig. 7:**
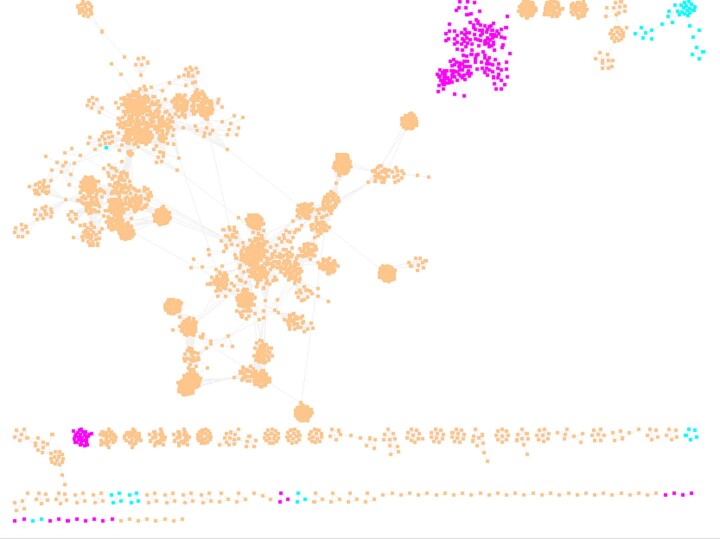
Gene-sharing networks produced via vCONTACT2 indicate that all ANME-1 mobile genetic elements (magenta) are well distinguished from the known haloarchaeal viruses, or Haloviruses, (blue) and other viruses with known hosts (orange).

ANME-1 genomes recovered in this study contained various CRISPR–Cas loci (Extended Data Fig. 6a), enabling the analysis of ANME-1-hosted mobile genetic elements (MGEs) through CRISPR spacer-based sequence mapping^33,34^ with additional stringent filters (see Methods and Supplementary Information). Mapping 20,649 unique ANME-1 CRISPR spacers to metagenomic assemblies from the Southern Pescadero and Guaymas Basins (Supplementary Table 7), and the metagenome-derived virus database IMG/VR v.3^35^ captured 76, 69 and 88 MGE contigs larger than 10 kb, respectively, totalling 233 ANME-1 MGEs (Fig. 4a, Extended Data Fig. 6b, Supplementary Tables 8 and 9 and Supplementary Data 1 and 2). Notably, all IMG/VR-derived ANME-1 MGEs originated from various Guaymas Basin-derived metagenomes. As previously found for the Asgard archaeal mobilome^34^, an apparent cross-site spacer-mobilome mapping indicates a substantial fraction of the ANME-1 mobilome has migrated across these sediment-hosted hydrothermal vent ecosystems in the Gulf of California, along with their hosts^23^ (Fig. 4a). Due to the apparent overlap of CRISPR repeats across diverse ANME-1 lineages, these spacers, and thus the host-MGE interactions, were not further assigned taxonomically to specific ANME-1 subclades. All MGEs identified in this study are distant from all other known viruses (Extended Data Fig. 7). A large fraction of these ANME-1 MGEs were found to be interconnected, forming one large complex gene-similarity network of 185 nodes and a medium-sized network of 28 nodes (Fig. 4b). The remaining 22 MGEs fell into seven small groups of two to three nodes, and seven singletons.

Based on the conservation of signature genes encoding viral structural proteins, we concluded that these MGEs encompass double-stranded DNA viruses belonging to at least four widely different virus assemblages characterized by different evolutionary histories and distinct virion morphologies (Fig. 4c). In particular, head-tailed viruses of the class Caudoviricetes (realm *Duplodnaviria*) encode characteristic HK97-fold MCPs and the large subunit of the terminase and portal proteins^36^; tailless icosahedral viruses of the realm *Varidnaviria* are characterized by double jelly-roll (DJR) MCPs^36^; viruses of the realm *Adnaviria* encode unique α-helical MCPs, which form claw-like dimers that wrap around the viral DNA forming a helical, rod-shaped capsid^37^; and all spindle-shaped viruses encode unique, highly hydrophobic α-helical MCPs^17^ (Supplementary Tables 9 and 10). In total, 16 candidate viral families were discovered in this study, including five families with representative complete genomes (Fig. 4c). We named these candidate virus families after Mayan gods, owing to their discovery in the Gulf of California hydrothermal vents off the coast of Mexico (see Methods for the etymology of the virus family names).

### Tailless icosahedral ANME-1 viruses

Tailless icosahedral viruses (*Varidnaviria*) infecting ANME-1 are distinguished from known viruses, with all 32 representatives unique to this study. They form three disconnected modules and, based on gene similarity analysis, represent three unreported viral families (Fig. 4c–f). Members of the Huracanviridae group encode single jelly-roll MCPs related to those conserved in the kingdom Helvetiavirae, whereas Chaacviridae and Ixchelviridae were unified within the order that we name Coyopavirales and do not encode MCPs with sequence homology to other known viruses. However, structural modelling of the candidate MCPs conserved in ‘Chaacviridae’ and ‘Ixchelviridae’ using AlphaFold2^38^ and RoseTTAFold^39^ revealed unambiguous similarity to the MCPs with a DJR fold (Fig. 4g). Phylogenetic analysis revealed that these DJR MCPs form three highly divergent groups, MCP-1–3 (Fig. 4g and h), with MCP-2 and MCP-3 containing an additional small beta-barrel that is predicted to point outwards from the capsid surface and likely mediate virus–host interactions.

Chaacviruses have linear dsDNA genomes with inverted terminal repeats and, accordingly, encode protein-primed family B DNA polymerases (pPolB). Chaacviruses display a remarkable genome plasticity; not only do these viruses encode two different variants of the DJR MCPs, MCP-1 and MCP-2, but their pPolBs belong to two widely distinct clades. Notably, the two MCP and two pPolB variants do not strictly coincide, suggesting multiple cases of recombination and gene replacement within the replicative and morphogenetic modules (Fig. 4d). Maximum-likelihood analysis of these divergent groups of pPolB sequences revealed relatedness to two separate clades of pPolBs encoded by Wyrdviruses, spindle-shaped viruses that target Asgard archaea^40^ (Fig. 4i). In addition to pPolB, upstream of the MCP gene, all chaacviruses encode a functionally uncharacterized protein with homologues in Asgard archaeal viruses of the Huginnvirus group, where they are also encoded upstream of the MCP genes^41^. This observation suggests a remarkable evolutionary entanglement between these ANME-1 and Asgard archaeal viruses, potentially facilitated by the ecological (that is, deep-sea vent ecosystems) rather than evolutionary proximity of the respective hosts.

### Viruses with unique structural and replicative features

**Fig. 5:**
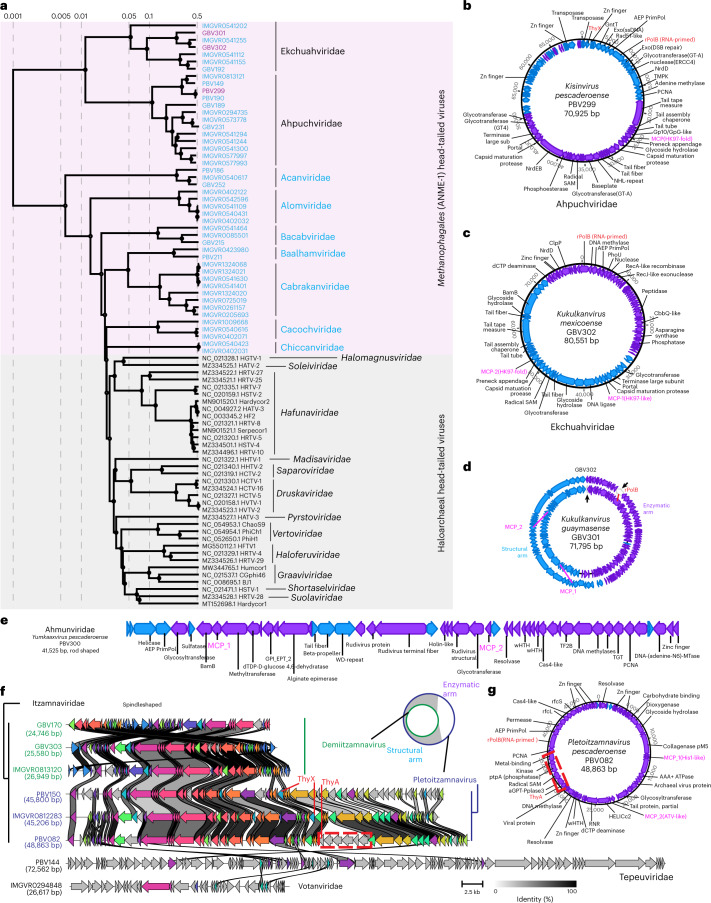
ANME-1 viral genomes encode complex structures. **a**, Evolutionary division between head-tailed viruses targeting ANME-1 and haloarchaea revealed by global proteome-based phylogenetic analyses. ANME-1 viruses with complete circular genomes are highlighted in purple, those with unconfirmed completeness are in blue. **b**,**c**, Genome organization and gene content of the complete genomes representing two families of ANME-1 head-tailed viruses Ahpuchviridae (**b**) and Ekchuahviridae (**c**). Blue and purple shading represents forward and reverse strands, respectively. MCP, PolB and ThyX genes are highlighted in pink and red. **d**, Circular alignment of the two genomes of ekchuahviruses. Black arrowheads indicate the original contig start/end sites in each assembly. **e**, Gene content of the complete linear genome of a representative of the rod-shaped virus family*Ahmunviridae*. **f**, Gene synteny of three families of spindle-shaped viruses targeting ANME-1, where complete, circularized genomes of Itzamnaviridae were found to occur in two genome sizes, where Demiitzamnavirus representatives align with a section of the larger Pletoitzamnavirus genomes (illustrated on the top right). Different colours indicate 76 different protein groups. Grey shading denotes singletons. The scale bar and percent identity shading are indicated in the bottom right. **g**, Gene content of the complete linear genome of a representative of the spindle-shaped virus family *Itzamnaviridae*. Dashed red box in (**f**) and (**g**) highlights an example of a multigene cluster insertion. In (**d**) and (**f**), the structural arm denotes the genome fraction where all viral structural genes reside; the enzymatic arm denotes the fraction where there are no structural genes and only enzyme-encoding genes reside.

**Extended Data Fig. 8:**
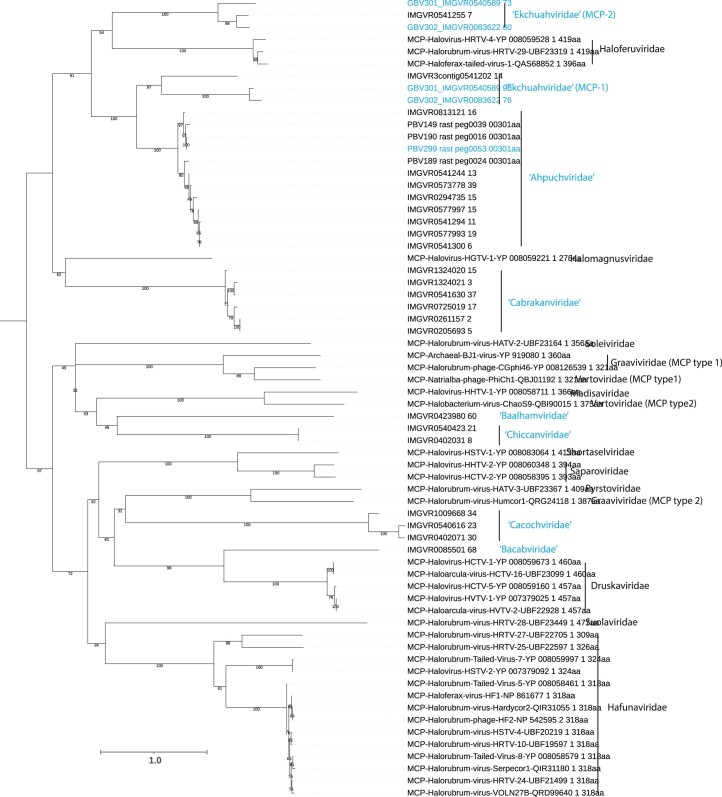
Maximum-likelihood analyses of MCPs encoded by head-tailed viruses. On the right: Blue, ANME-1 virus families; black, haloarchaeal virus families. MCPs from complete genomes of ANME-1 viruses are indicated in blue, and their respective families in bold.

**Extended Data Fig. 9:**
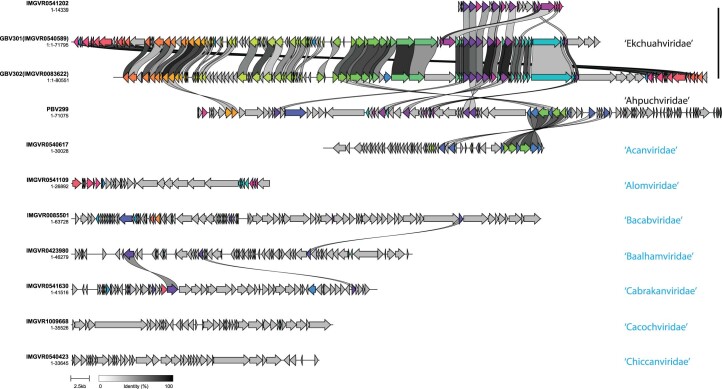
Sequence alignment of representatives of ANME-1 viruses. Colors indicates protein families encoded by at least 2 representative viral genomes here. Gray indicates singleton proteins without apparent homologs. Scale bars for protein identity scores and genome sizes are indicated at the bottom. Viral contig names and sizes are indicated on the left side and their respective family names are indicated on the right.

The head-tailed viruses targeting ANME-1 encode the typical morphogenetic toolkit shared between all members of the Caudoviricetes, including the HK97-fold MCP, portal protein, large subunit of the terminase and various tail proteins^42^. MCP phylogeny indicates a shared ancestry for the structural components of the viruses of ANME-1 and haloarchaea, which are related at the phylum level (Extended Data Fig. 8). However, global proteome-based phylogeny^43^ revealed a clear division between ANME-1 and haloarchaeal head-tailed viruses (Fig. 5a). This result suggests that although these viruses encode related core proteins for virion formation, as suggested by their interspersed MCP phylogenetic positions (Extended Data Fig. 8), the overall proteome contents of ANME-1 and haloarchaeal viruses differ considerably, likely reflecting the adaptation to their respective hosts and ecological contexts. Based on the minimum genetic distances between halovirus families and cross-genome comparisons (Extended Data Fig. 9), we propose nine candidate Caudoviricetes families. Viruses in these families exhibit little proteome overlap with each other (Extended Data Fig. 9), further illustrating the vast genetic diversity of ANME-1 head-tailed viruses.

Ekchuahviridae and Ahpuchviridae are represented by ANME-1 viruses with complete 70–80 kb genomes and in the proteomic tree form sister clades outside of the three orders of haloviruses, forming an independent order that we name Nakonvirales (Fig. 5a). The ahpuchviruses PBV299 (70.9 kb, complete, Fig. 5b) and IMGVR0573778 (74.8 kb, near complete) each encode one copy of MCP, whereas the two ekchuahviruses GBV302 (80.6 kb, complete, Fig. 5c) and GBV301 (71.8 kb, complete)^35,44^ each encode two MCP copies. This is unique among other known Caudoviricetes targeting haloarchaea and ANME-1. We can exclude an assembly artefact, because the initial assemblies of the two ekchuahviruse*s* were found to have a circular alignment with each other (Fig. 5d). Both MCP genes are accompanied by cognate capsid maturation protease genes, whereas all other virion morphogenetic proteins are encoded as single copy genes (Fig. 5c). MCP-1 is likely ancestrally conserved, whereas MCP-2 appears horizontally transferred from haloferuviruses. Their large phylogenetic distance suggests a long coexistence and coevolution in ekchuahviruses.

The coexistence of two divergent MCP genes is also found in members of putative rod-shaped viruses within the family ‘Ahmunviridae’, which we propose including into the class Tokiviricetes (realm *Adnaviria*)^37^ within a monotypic order ‘Maximonvirales’ (Fig. 5e), and viruses with predicted spindle-shaped morphology, the ‘Itzamnaviridae’ (Fig. 5f–g). These two previously undescribed clades of viruses are represented by complete linear genomes with inverted terminal repeats and circular genomes, respectively. This contrasts another spindle-shaped ANME-1 virus, the tepeuvirus PBV144, which has the largest genome (72.6 kb, not yet circularized) but only one MCP. The coexistence of divergent MCPs is unusual among Caudoviricetes, but has been previously documented for the head-tailed T4 phage, whose MCPs respectively form hexameric and pentameric capsomers, with the latter occupying the fivefold icosahedral vertices^45^. Dual-MCP rod-shaped viruses either form a functional MCP heterodimer^37,46^ or use only one copy for virion formation^47^. It is thus yet unclear how coexisting MCP genes contribute to the capsid architecture of ANME-1 viruses.

### Viral auxiliary functions and virus-driven ANME-1 evolution

The large genomes of head-tailed and spindle-shaped viruses of ANME-1 exhibit strong clustering of functionally related genes: one half of the viral genome contains all structural genes, whereas the other half encodes diverse enzymes involved in DNA synthesis and modification and various metabolic and defence functions (Fig. 5b–d,f,g). Notably, the entire approximately 20 kb replicative and metabolism module is missing from the circular genomes of demiitzamnaviruses. Cross-genome alignments revealed a larger variation in gene content for the enzymatic arms in both head-tailed and spindle-shaped viruses, frequently in the form of multigene cluster insertions (Fig. 5f and Extended Data Fig. 9). Head-tailed Ekchuahviridae and Ahpuchviridae and spindle-shaped Pletoitzamnavirus and Tepeuviridae encode RNA-primed family B DNA polymerases, which are commonly encoded by dsDNA viruses with larger genomes^48^. The structural-enzymatic arm split thus resembles the core- and pan-genomes of microbes, allowing versatile interactions between these viruses and their ANME-1 hosts (Supplementary Table 10). For example, head-tailed and spindle-shaped viruses contain auxiliary metabolic genes involved in nucleotide and amino acid metabolisms (NrdD, QueCDEF and asparagine synthase), carbon anabolism (PEPCK and GntT) and phosphate and sulfur anabolism (PhoU and PAPS) (see Supplementary Table 11 and Supplementary Information).

**Fig. 6:**
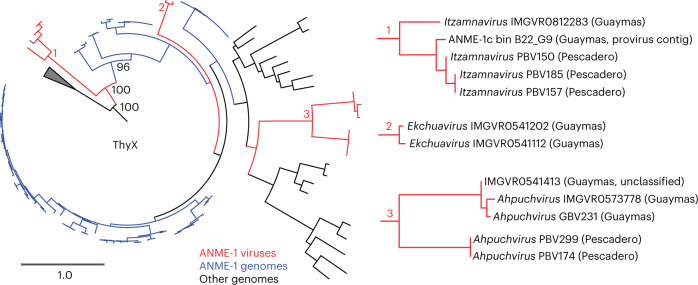
A viral origin of thymidylate synthase in ANME-1. Maximum-likelihood analysis of ThyX related to ANME-1 encoded ThyX proteins, with expanded views of ThyX from ANME-1 viruses on the right. Legends for the branch colours for ThyX from ANME-1 viruses and ANME-1 genomes are indicated below the main phylogenetic tree.

**Extended Data Fig. 10:**
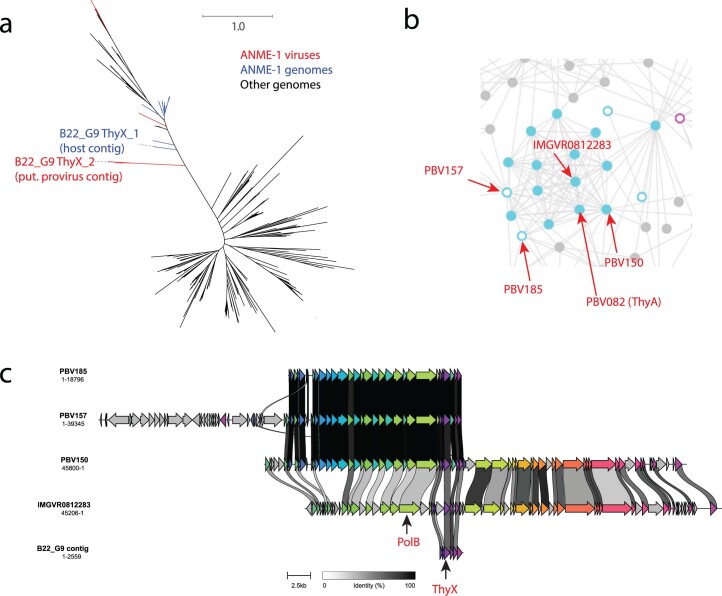
Spindle-shaped viruses encode ThyX at the root of ANME-1 ThyX. **a**, unrooted phylogeny suggests that ANME-1 *thyX* may have evolved from *thyX* genes originated in ANME-1 viruses. The two versions of of ThyX in the ANME-1c bin B22_G9 are highlighted. **b**, Distribution of ThyX gene in various spindle-shaped viruses indicated on the gene-sharing network. **c**, Sequence alignment showing the conservation and variation of gene content around the *thyX* gene in the genomes of spindle-shaped viruses.

Our analysis of viral auxiliary metabolic genes also suggested the involvement of viruses in the ancestral metabolic diversification of ANME-1. Specifically, the detection of genes encoding ThyX^49^, which catalyses dUMP methylation into dTMP and likely boosts host thymidine synthesis during viral production, in head-tailed ahpuchviruses and ekchuahviruses and in spindle-shaped pletoitzamnaviruses (Fig. 5b,f). This coincides with the presence of *thyX* in the ANME-1 host, which unlike other ANME lineages and short-chain alkane-oxidizing archaea, do not encode the non-homologous thymidylate synthase gene, *thyA*^2^ (Fig. 1). The dichotomous distribution of the functional analogues *thyA/thyX* is prevalent across microbes and, notably, in itzamnaviruses, *thyX* and *thyA* may exist in different members (Fig. 5f,g). Phylogenetic analysis of the ThyX show it was encoded by ANME-1 and their viruses form a distinct clade distant from those encoded by bacteria, archaea and other Caudoviricetes (Fig. 6 and Extended Data Fig. 10a,b). Strikingly, ThyX encoded by itzamnaviruses form a well-supported monophyletic group at the base of this divergent clade and the deep-branching ANME-1c encode ThyX that belong to the second deepest branch. Notably, the Guaymas Basin ANME-1c bin (B22_G9) contains both a genomic *thyX*, and *thyX* encoded by a partial itzamnavirus-derived contig (Fig. 6 and Extended Data Fig. 10). Ekchuahviruses and ahpuchviruses likely acquired *thyX* independently at a later stage.

The above analyses suggest *thyX* was first acquired by spindle-shaped ANME-1 viruses, then transmitted into the common ancestors of ANME-1, displacing *thyA*. Due to higher promiscuity of viral DNA polymerases and the intense arms race, viral genes are known to evolve rapidly^50^, which is in line with the extreme divergence of the ANME-1/viral *thyX* from the canonical clade.

## Discussion

In this study, metagenomic characterization of a recently discovered hydrothermal vent environment in the Southern Pescadero Basin led to the expansion of the known ANME-1 diversity to include ANME-1c and their viruses. ANME-1c is a deep-branching family that so far has only been detected in high temperature hydrothermal environments. Comparative genomics indicates an evolutionary continuum within the class *Syntrophoarchaeia*, because ANME-1c retained various ancestral features also found in Syntrophoarchaeales and Alkanophagales, including hydrogenases. The phylogeny of these hydrogenases is congruent with the genome phylogeny indicating an apparent vertical inheritance and differential loss of these genes in ANME-1, suggesting these hydrogenases have a nonobligatory physiological role, but may confer a longstanding selective advantage.

Our study also uncovered a putative viral source of the ANME-1-specific thymidylate synthase gene *thyX* that replaced the functional analogue *thyA* gene. ThyX differs from ThyA in its use of NADPH as the electron donor when transferring the methyl group from the C_1_ intermediate H_4_MPT = CH_2_ to dUMP to yield dTMP, without oxidizing the H_4_MPT moiety^2^. H_4_MPT is a core cofactor constantly recycled through the Wood-Ljungdahl pathway that fuels ANME-1 anabolism^2^; NADPH abundance is highly dependent on the type of host energy metabolism and redox state^51^. The virus-induced ThyA to ThyX transition may have played a role in the metabolic diversification and subsequent ecological expansion of the ANME-1 ancestors. C_1_ anabolism appears to be more divergent across ANME lineages than C_1_ energy metabolism^2^, which may have also originated from viruses and other MGEs.

The expansive virome of ANME-1, as discovered by this study, is distant from all known viruses, forming 16 previously undescribed families and at least three unreported orders. They are characterized by many unique structural and replicative features, substantially expanding our appreciation of the archaeal virus diversity and their ecological importance. These findings open the door for targeted culture-dependent and culture-independent exploration of ANME virus–host interactions that are expected to play a critical role in the biogeochemical cycling^19^ in these productive methane-driven ecosystems^1^.

While this paper was in review, a paper describing the enrichment of a strain of ‘*Candidatus* Methanoxibalbensis ujae’ under thermophilic methanotrophic conditions was published^52^.

## Methods

### Sampling and incubation

Four rock samples were collected from the 3.7 km-deep Auka vent field in the Southern Pescadero Basin (23.956094N, 108.86192W)^20,23^. Sample NA091.008 was collected in 2017 on cruise NA091 with the Eexploration vessle Nautilus and incubated as described previously^34^. Samples 12,019 (S0200-R1), 11,719 (S0193-R2) and 11,868 (S0197-PC1), the latter representing a lithified nodule recovered from a sediment push core, were collected with Remotely operated vehicle SuBastian and Research vessel Falkor on cruise FK181031 in November 2018. These samples were processed shipboard and stored under anoxic conditions at 4 °C for subsequent incubation in the laboratory. In the laboratory, rock samples 12,019 and 11,719 were broken into smaller pieces under sterile conditions, immersed in N_2_-sparged sterilized artificial sea water and incubated under anoxic conditions with methane, as described previously for NA091.008 (ref. 34). Additional sampling information can be found in Supplementary Table 1. Mineralogical analysis by X-ray Powder Diffraction (XRD) identified barite in several of these samples, collected from two locations in the Auka vent field, including on the western side of the Matterhorn vent (11,719, NA091.008), and one oil-saturated sample (12,019) recovered from the sedimented flanks from the southern side of Z vent. Our analysis also includes metagenomic data from two sediment cores from the Auka vent field (DR750-PC67 and DR750-PC80) collected in April 2015 with the ROV Doc Ricketts and R/V Western Flyer (MBARI2015), previously published (ref. 23).

### Fluorescence in situ hybridization

Samples were fixed shipboard using freshly prepared paraformaldehyde (2 vol% in 3× Phosphate Buffer Solution (PBS), EMS15713) at 4 °C overnight, rinsed twice using 3× PBS, and stored in ethanol (50% in 1× PBS) at −20 °C until processing. Small pieces (<1 cm^3^) of the mineral sample NA091.008 were gently crushed in a sterile agate mortar and pestle in a freshly prepared, filter sterilized 80% ethanol – 1× PBS solution. About 500 μl of the resulting mixture was sonicated three times in 15 second bursts on a Branson Sonifier W-150 ultrasonic cell disruptor (level 3) on ice with a sterile remote-tapered microtip probe inserted into the liquid. Cells were separated from the mineral matrix using an adapted protocol of density separation using Percoll (Sigma P4937)^7^. The density-separated cells were filtered on 25 mm polycarbonate filters with a pore size of 0.22 μm (Millipore GTTP2500), and rinsed using 1× PBS. Fluorescence in situ hybridizations were carried out as described previously^7^ using a 1:1 mixture of an ANME-1 targeted probe (ANME-1-350^9^ labelled with Cy3) and the general bacterial probe mix EUB338 I-III (https://probebase.csb.univie.ac.at/), labelled with Alexa-488 in a 35% formamide solution (VWR EM-FX0420-8). Hybridized samples were imaged using a ×100 objective using a Zeiss Elyra structured illumination microscope with the Zen Black software.

### DNA extraction and sequencing

DNA extraction from the mineral samples followed previously published protocols^34^. Metagenomic analysis from the extracted genomic DNA was outsourced to Quick Biology (Pasadena, CA) for library preparation and sequencing. Libraries were prepared with the KAPA Hyper plus kit using 10 ng of DNA as input. This input was subjected to enzymatic fragmentation at 37 °C for 10 min. After end repair and A-tailing, the DNA was ligated with an IDT adaptor (Integrated DNA Technologies Inc.). Ligated DNA was amplified with KAPA HiFi HotStart ReadyMix (2×) for 11 cycles. Post-amplification cleanup was performed with 1× KAPA pure beads. The final library quality and quantity were analysed and measured by Agilent Bioanalyzer 2100 (Agilent Technologies) and Life Technologies Qubit 3.0 Fluorometer (Life Technologies), respectively. Finally, the libraries were sequenced using 150 bp paired-end reads on Illumina HiSeq4000 Sequencer (Illumina Inc.). After sequencing, primers and adaptors were removed from all libraries using bbduk (https://sourceforge.net/projects/bbmap/) with mink = 6 and hdist = 1 as trimming parameters, and establishing a minimum quality value of 20 and a minimal length of 50 bp. For nanopore sequencing of incubated samples, DNA was amplified using multiple displacement amplification with the QIAGEN REPLI-g Midi kit before library preparation. Oxford Nanopore sequencing libraries were constructed using the PCR-free barcoding kit and were sequenced on PromethION platform by Novogene Inc.

### Metagenomic analysis

The sequencing reads from unincubated rocks were assembled individually and in a coassembly using SPAdes v.3.12.0 (ref. 53). From the de-novo assemblies, we performed manual binning using Anvio v.6 (ref. 54). We assessed the quality and taxonomy affiliation from the obtained bins using GTDB-tk v.1.5.0 (ref. 55) and checkM v.1.13 (ref. 56). Genomes affiliated to ANME-1 and Syntrophoarchaeales were further refined via a targeted-reassembly pipeline. In this pipeline, the original reads were mapped to the bin of interest using bbmap (https://sourceforge.net/projects/bbmap/), then the mapped reads were assembled using SPAdes and the resulting assembly was filtered discarding contigs below 1,500 bp. This procedure was repeated during several rounds (between 11 and 50) for each bin, until we could not see an improvement in the bin quality. Bin quality was assessed using the checkM and considering the completeness, contamination (<5%), N50 value and number of scaffolds. The resulting bins were considered as MAGs. The sequencing reads for the incubated rocks 12,019 and 11,719 were assembled as described previously for NA091.R00834. Additionally, the assembly of 12,019 was then scaffolded using Nanopore reads through two iterations of LRScaf v.1.1.10 (ref. 57). The final assemblies were binned using metabat2 v.2.15 (ref. 58) using the default setting. Automatic metabolic prediction of the MAGs was performed using prokka v.1.14.6 (ref. 59) and curated with the identification of PFAM and TIGRFAM profiles using HMMER v.3.3 (hmmer.org), KEGG orthologs with Kofamscan^60^ and of COGs and arCOGs motifs^61^. To identify multiheme cytochromes in our genomes, we searched the motif CXXCH across the amino acid sequences predicted for each MAG. Similar metabolic predictions were carried out with publicly available ANME-1 and Syntrophoarchaeales genomes to compare the metabolic potential of the whole ANME-1 order. A list of the genomes used in this study can be found in Supplementary Table 2. For the comparison of different genomic features among the ANME-1 genomes, we searched for specific proteins using the assigned COGs, arCOGs and KEGG identifiers (Supplementary Table 5).

### Genomic relative abundance analysis

We used the software coverM v.0.5 (https://github.com/wwood/CoverM) to calculate the genomic relative abundance of the different organisms of our samples, using all the MAGs we have extracted from our metagenomic analysis. We ran the software with the following additional parameters for dereplication (‘–dereplication-ani 95–dereplication-prethreshold-ani 90–dereplication-precluster-method finch’). Results were visualized in R v.4.2.1.

### OGT analysis

We calculated the OGT for all ANME-1 and Syntrophoarchaeales MAGs included in our analysis (Supplementary Table 2) using the OGT_prediction tool described in Sauer and Wang^24^ with the regression models for Archaea excluding rRNA features and genome size.

### Analysis of hydrogenase operons

Because only two of the five genomes of ‘*Candidatus* Methanospirare jalkutatii’ have an operon encoding a hydrogenase, we performed additional analysis to better understand this intraspecies distribution. On the one hand, we mapped the metagenomic reads from samples with genomes of ‘*Candidatus* Methanospirare jalkutatii’ (12019, FW4382_bin126, NA091.008, PR1007, PR1031B) to the MAGs containing the hydrogenase operon (FW4382_bin126, NA091.008_bin1) to check if reads mapping this operon are also present in samples from where the MAGs without the hydrogenase were recovered. For mapping the reads, we used bowtie2 v.2.4.2 (ref. 62) then transformed the sam files to bam using samtools (http://www.htslib.org/) and extracted the coverage depth for each position. Additionally, we performed a genomic comparison of the genomes with a hydrogenase operon (FW4382_bin126, NA091.008_bin1) with the genome FWG175 that was assembled into a single scaffold. For this, we used the genome-to-genome aligner Sibelia v.3.0.7 (ref. 63) and we visualized the results using Circos (http://circos.ca/).

### Phylogenetic analysis

For the phylogenomic tree of the ANME-1 MAGs, we used the list of genomes present in Supplementary Table 2. As marker genes, we used 31 single copy genes (Supplementary Table 5) that we extracted and aligned from the corresponding genomes using anvi-get-sequences-for-hmm-hits from Anvio v.6 (ref. 54) with the parameters ‘–return-best-hit–max-num-genes-missing-from-bin 7–partition-file’. Seven genomes missed more than seven marker genes and were not used for the phylogenomic reconstruction present in Fig. 1 (ANME-1 UWMA-0191, Syntrophoarchaeum GoM_oil, ANME-1 ERB7, ANME-1 Co_bin174, ANME-1 Agg-C03, PB_MBMC_218, FW4382_bin035). The concatenated aligned marker gene set was then used to calculate a phylogenomic tree with RAxML v.8.2.12 (ref. 64) using a partition file to calculate differential models for each gene the following parameters ‘-m PROTGAMMAAUTO -f a -N autoMRE -k.’ The tree was then visualized using iTol^65^. For the clustering of the MAGs into different species, we dereplicated the ANME-1 MAGs using dRep v.2.6.2 with the parameter ‘-S_ani 0.95’ (ref. 66). A smaller phylogenomic tree was calculated with the genomes containing hydrogenase genes (Fig. 3). For this tree we also used Anvio v.6 and RAxML v.8.2.12 with the same parameters but excluding the flag ‘—max-num-genes-missing-from-bin’ from the anvi-get-sequences-for-hmm-hits command to include in the analysis those genomes with a lower number of marker genes that still contain hydrogenase genes (PB_MBMC_218, FW4382_bin035, ANME-1 UWMA-0191).

The 16S rRNA gene phylogenetic tree was calculated for the 16S rRNA genes predicted from our genome dataset that were full length. We included these full-length 16S rRNA genes in the SILVA_132_SSURef_NR99 database^67^ and with ARB v.6.1 (ref. 68) we calculated a 16S phylogenetic tree using the maximum-likelihood algorithm RAxML with GTRGAMMA as the model and a 50% similarity filter. In total, 1,000 bootstrap analyses were performed to calculate branch support values. The tree with the best likelihood score was selected.

For the construction of the hydrogenase phylogenetic tree (Supplementary Table 6), we used the predicted protein sequence for the large subunit of the NiFe hydrogenase present in the genomes of our dataset (Supplementary Table 2), a subset of the large subunit hydrogenases present in the HydDB database^30^ and the predicted hydrogenases present in an archaeal database using the COG motif for the large NiFe hydrogenase (COG0374) with the Anvio v.6 software. For the mcrD gene phylogeny, we used the predicted protein sequences of mcrD in the ANME-1c genomes and in the previously mentioned archaeal database with the TIGR motif TIGR03260.1 using also the Anvio v.6 software. The list of genomes from the archaeal database used in the analysis can be found in Supplementary Table 6. For both phylogenies, the protein sequences for the analysis were aligned using clustalw v.2.1 with default settings^69^. The aligned file was used to calculate a phylogenetic tree using RAxML v.8.2.12 (ref. 64) with the following parameters ‘-m PROTGAMMAAUTO -f a -N 100 –k’. The tree was then visualized using iTol^65^.

For the distribution and phylogenetic analysis of MCP and pPolB, known sequences encoded by various bacterial and archaeal viruses were used to build a Hidden Markov Model (HMM) via hmmer v.3.3.2. The HMM was then used to capture the corresponding components in proteomes of ANME-1 viruses and other MGEs. All sequences were then aligned using MAFFT v.7.475 (ref. 70) option linsi and trimmed using trimAl v1.4.1 (ref. 71) option gappyout for pPolB and 20% gap removal option for MCP. Maximum-likelihood analyses were carried out through IQtree v.2.1.12 (ref. 72) using model finder and ultrafast bootstrap with 2,000 replicates. The phylogenetic tree was visualized and prepared using iTol^65^.

For the distribution and phylogenetic analysis of ThyX, all ThyX sequences annotated by EggNOG mapper^73^ v.2 in the genomes of ANME-1 and their MGEs were used to create a HMM as described above, and used to search for close homologues in the GTDB202 database, IMGVR V.3 database and again in the proteomes and ANME-1 and their MGEs in this study. This yielded 261 sequences, which was then aligned and phylogenetically analysed as for pPolB.

### CRISPR analysis

The CRISPR–Cas systems from the ANME-1 genomes and various metagenomic assemblies were annotated using CRISPRCasTyper v.1 (ref. 33). CRISPR spacer mapping on MGEs was carried out as previously described^34^ with the following modifications. To filter out unreliable sequences that may have arisen during MAG binning, we took a conservative measure of only retaining CRISPR repeats identified in at least three ANME-1 contigs. We additionally analysed the CRISPR repeats found in the Alkanophagales sister clade to ANME-1 using the same approach, which were found to have no overlap with the ANME-1 CRISPR repeats. To avoid accidental mapping to unrelated MGEs, we applied a second stringent criteria of only retaining MGEs with at least three ANME-1 protospacers. MGEs larger than 10 kb were retained for further analyses in this study.

### MGE network analysis and evaluation

Open reading frames in all CRISPR-mapped MGE contigs were identified using the PATRIC package^74^. Gene similarity network analyses were done using vCONTACT v.2.0 (ref. 75) using the default reference (RefSeq202), with head-tailed viruses targeting haloarchaea and methanogens added as extra references^42^. Inverted and direct terminal repeats were detected using CheckV v.1.01. and the PATRIC package to determine genome completeness. Clustering confidence were obtained with default setting as described in ref. 75, where the *P* value was obtained via a one-sided Mann–Whitney *U* test and the topology confidence is obtained by multiplying the quality score of the subcluster and the *P* value.

### MGE annotation and virus identification

MGE proteomes are annotated using sensitive HMM profile-profile comparisons with HHsearch v.3.3.2 (ref. 76) against the following publicly available databases: Pfam 33.1, Protein Data Bank (25 March 2021), CDD v.3.18, PHROG and uniprot_sprot_vir70 (9 February 2021)^77^. Putative MCP of Chaacviridae and Ixchelviridae could not be identified using sequence similarity-based approaches. Thus, the candidate proteins were subjected to structural modelling using AlphaFold2 (ref. 38) and RoseTTAFold v.1.1.0 (ref. 39). The obtained models were visualized using ChimeraX^78^ and compared with the reference structure of the MCP of corticovirus PM2 (PDB id: 2vvf). The contigs containing identifiable viral structural proteins are described as viruses. The remaining contigs are described as unclassified MGEs, including circular elements that are most likely plasmids of ANME-1 and possible viruses enveloped by yet unknown structural proteins.

### Genome-scale virus comparisons

The viral genomes were annotated using Prokka v.1.14.6 (ref. 59) to produce genbank files. Select genbank files were then analysed using Clinker v.0.0.23 (ref. 79) to produce the protein sequence clustering and alignments. Proteome-scale phylogeny for the head-tailed viruses were carried out via the VipTree server^43^.

### Etymology

#### Descriptions of proposed ANME-1c family and species

**Family** ‘***Candidatus*** **Methanospirareceae**’. N.L. neut. n. methanum methane; N.L. pref. methano-, pertaining to methane; L.v. spirare, to breathe. Proposed classification: class Methanomicrobia, order ‘*Candidatus* Methanophagales’. The type species and strain is ‘*Candidatus* Methanospirare jalkutatii’ FWG175.

‘***Candidatus*** **Methanoxibalbensis ujae**’. N.L. neut. n. methanum methane; N.L. pref. methano-, pertaining to methane; N.L. adj. xibalbensis, from the place called Xibalba, the Mayan word for the underworld; N.L. neut. n. *Methanoxibalbensis* methane-cycling organism present in deep-sea hydrothermal sediments; N.L. neut. adj. ujae, from the word ujá, meaning rock in Kiliwa, an indigenous language of the native peoples of Baja California, referring to the high abundance of this species in rock samples. Proposed classification: class Methanomicrobia, order ‘*Candidatus* Methanophagales’, family ‘*Candidatus* Methanoxibalbaceae’, genus ‘*Candidatus* Methanoxibalbensis’.

The material type is the genome designated NA091.008_bin2 (GCA_026134085.1), a MAG comprising 1.99 Mbp in 86 scaffolds. The MAG was recovered from mineral sample (NA091.008) from the hydrothermal environment of South Pescadero Basin.

‘***Candidatus*** **Methanospirare jalkutatii**’. N.L. neut. n. methanum methane; N.L. pref. methano-, pertaining to methane; L.v. spirare, to breathe; N.L. neut. n. Methanospirare methane-breathing organism; N.L. masc. n. jalkutatii, a mythical dragon from stories of the indigenous Pa ipai people from Northern Baja, California. This dragon inhabited a beautiful place made of rocks and water similar to the Auka hydrothermal vent site. Proposed classification: class Methanomicrobia, order ‘*Candidatus* Methanophagales’, family ‘*Candidatus* Methanoxibalbaceae’, genus ‘*Candidatus* Methanospirare’.

The material type is the genome designated FWG175 (CP110382.1), a single-scaffolded MAG comprising 1.62 Mbp in one circular scaffold. This MAG was recovered from a methane-fed incubation of the mineral sample 12,019 retrieved from the hydrothermal environment of South Pescadero Basin.

### Proposed classification of ANME-1 viruses

The order Coyopavirales is proposed within the existing class Tectiliviricetes, after Coyopa, the god of thunder in Mayan mythology. It contains tailless icosahedral viruses with previously unreported class of DJR MCPs and little proteome overlap with known viruses. The family Chaacviridae is proposed within Coyopavirales, after Chaac, the god of death in the Mayan mythology. It is characterized by a uniform 10–11 kb genome and a gene encoding protein-primed family B DNA polymerase (pPolB). We propose the genus names *Homochaacvirus* and *Antichaacvirus* (from homo, for same in Greek, and anti, for opposed in Greek, to emphasize the inversion of a gene module including the *pPolB* gene). Six complete genomes of chaacviruses have been obtained: Methanophagales virus PBV304 (OP548099) within sepcies *Homochaacvirus pescaderoense*, Methanophagales virus PBV305 (OP548100) within species *Homochaacvirus californiaense*, Methanophagales virus GBV261*,* Methanophagales virus GBV265*,* Methanophagales virus GBV275 and Methanophagales virus PBV266 (OP413841) within species *Antichaacvirus pescaderoense*. The candidate family Ixchelviridae is proposed within Coyopavirales, after Ix Chel, goddess of midwifery and medicine in the Mayan mythology. Ixchelviridae is represented by Pescadero Basin viruses PBV176 and PBV180, with assembly completeness unknown.

Candidate family Huracanviridae is proposed without higher-level ranking classification, after Hurancan, god of wind, storm and fire in Mayan mythology. It contains tailless icosahedral viruses with single jelly-roll MCPs. It is represented by Pescadero Basin viruses PBV264 and PBV238, with assembly completeness undetermined.

The order Nakonvirales is proposed within Caudoviricetes, after Nakon, the most powerful god of war in Mayan mythology. It contains head-tailed viruses with around 80 kb genomes and HK97-fold MCPs. The family Ahpuchviridae (after Ah Puch, the god of death in the Mayan mythology) includes one genus, *Kisinvirus*, (after Kisin, another Mayan god of death) and is represented by a single virus, Methanophagales virus PBV299 (OP413838) within species *Kisinvirus pescaderoense*. The family Ekchuahviridae (after Ek Chuah, the patron god of warriors and merchants in Mayan mythology), is represented by one genus, *Kukulkanvirus* (after Kukulkan, the war serpent in the Mayan mythology). It includes Methanophagales virus GBV301 (OP880252) within species *Kukulkanvirus guaymasense* and Methanophagales virus GBV302 (OP880253) within species *Kukulkanvirus mexicoense*, each encoding two divergent HK97-fold MCPs with their own capsid maturation proteases.

Seven other candidate families of head-tailed viruses are proposed without complete genome representatives. They form a phylogenetic cluster sister to Haloviruses (Fig. 5a), and according to the phylogenetic classifications of the latter, likely form multiple unclassified order-level clades. These candidate families are Acanviridae, Alomviridae, Bacabviridae, Baalhamviridae, Cabrakanviridae, Cacochviridae and Chiccanviridae, all named after gods in Mayan mythology.

The order Maximonvirales is proposed within Tokiviricetes, after Maximon, a god of travellers, merchants, medicine men/women, mischief and fertility in Mayan mythology. It contains rod-shaped viruses of a single family Ahmunviridae (after Ah Mun, the god of agriculture in Mayan mythology) with a single genus *Yumkaaxvirus* (after Yum Kaax, the god of the woods, the wild nature and the hunt in Mayan mythology). It is represented by the complete linear genome of Methanophagales virus PBV300 (OP413840) within species *Yumkaaxvirus pescaderoense*.

The family Itzamnaviridae is named after Itzamna, lord of the heavens and night and day in Mayan mythology. It contains spindle-shaped viruses that differ in genome sizes and are subdivided into two genera, which we propose naming *Demiitzamnavirus* and *Pletoitzamnavirus* (after demi*-* for half or partial, derived via French from Latin dimedius and pleto for full in Latin). They are respectively represented by complete genomes of Methanophagales virus GBV170 within species *Demiitzamnavirus guaymasense*, Methanophagales virus GBV303 (OP880254) within species *Demiitzamnavirus mexicoense* and Methanophagales virus PBV082 (OP413839) within species *Pletoitzamnavirus pescaderoense*.

Candidate families *Tepeuviridae* and *Votanviridae*, named after a skye god Tepeu and a legendary ancestral deity Votan, respectively, are proposed for two additional new clades of spindle-shaped viruses. Their genome representatives Tepeuvirus PBV144 and Votanvirus IMGVR0294848 are not yet circularized and are thus incomplete.

### Reporting summary

Further information on research design is available in the Nature Portfolio Reporting Summary linked to this article.

## Supplementary information

Supplementary Information Brief discussion of some of the results of the paper: metabolic features of ANME-1c and viral genes.

Reporting Summary

Peer Review File

Supplementary Tables 1–12 12 Supplementary Tables with additional information.

Supplementary Data 1 Fasta file containing the CRISPR spacer sequences found in this study.

Supplementary Data 2 Fasta file containing ANME-1-specific MGEs from Guaymas and Pescadero Basins.

## Data Availability

Raw metagenome reads, assembled metagenome bins and virus sequence data are available in the NCBI database under BioProject accession numbers PRJNA875076 and PRJNA721962. Complete ANME-1 virus genomes representing new viral taxa can be found on GenBank under accession numbers OP413838, OP413839, OP413840, OP413841, OP548099, OP548100, OP880252, OP880253 and OP880254. CRISPR spacer sequences of ANME-1 and all genomic sequences of ANME-1 MGEs are also provided as supplementary data. For virus genomic analysis the following databases were used in this study: Protein Data Bank (namely, the major capsid protein of phage PM2, PDB id: 2vvf; https://www.rcsb.org/structure/2vvf), CDD v3.18, PHROG (https://phrogs.lmge.uca.fr/) and uniprot_sprot_vir70 (09/02/2021).
