# Supplementary material for: Evolutionary diversification of methanotrophic ANME-1 archaea and their expansive virome
Source: Nat Microbiol. 2023 Jan 19;8(2):231–45. doi: 10.1038/s41564-022-01297-4 (PMC9894754; doi:10.1038/s41564-022-01297-4)
Supplement: Supplementary file 2 — Reporting Summary [file 41564_2022_1297_MOESM2_ESM.pdf]

nature portfolio

Corresponding author(s): Rafael Laso-Pérez & Victoria Orphan

Last updated by author(s): 27/10/2022

Reporting Summary

Nature Portfolio wishes to improve the reproducibility of the work that we publish. This form provides structure for consistency and transparency in reporting. For further information on Nature Portfolio policies, see our Editorial Policies and the Editorial Policy Checklist.

Statistics

For all statistical analyses, confirm that the following items are present in the figure legend, table legend, main text, or Methods section.

n/a	Confirmed	
☒	☐	The exact sample size (*n*) for each experimental group/condition, given as a discrete number and unit of measurement
☒	☐	A statement on whether measurements were taken from distinct samples or whether the same sample was measured repeatedly
☒	☐	The statistical test(s) used AND whether they are one- or two-sided<br>*Only common tests should be described solely by name; describe more complex techniques in the Methods section.*
☒	☐	A description of all covariates tested
☒	☐	A description of any assumptions or corrections, such as tests of normality and adjustment for multiple comparisons
☐	☒	A full description of the statistical parameters including central tendency (e.g. means) or other basic estimates (e.g. regression coefficient) AND variation (e.g. standard deviation) or associated estimates of uncertainty (e.g. confidence intervals)
☒	☐	For null hypothesis testing, the test statistic (e.g. *F*, *t*, *r*) with confidence intervals, effect sizes, degrees of freedom and *P* value noted<br>*Give P values as exact values whenever suitable.*
☒	☐	For Bayesian analysis, information on the choice of priors and Markov chain Monte Carlo settings
☒	☐	For hierarchical and complex designs, identification of the appropriate level for tests and full reporting of outcomes
☒	☐	Estimates of effect sizes (e.g. Cohen's *d*, Pearson's *r*), indicating how they were calculated

Our web collection on statistics for biologists contains articles on many of the points above.

Software and code

Policy information about availability of computer code

Data collection	Zen black version ELYRA was used for the acquisition of fluorescent images on Zeiss microscope.
Data analysis	SPAdes v. 3.12.0; Anvio v. 6; GTDB-tk v.1.5.0; checkM v.1.1.3; LRScaf v1.1.10; metabat2 v2.15; prokka v. 1.14.6; HMMER v.3.3.2; Kofamscan; coverM v. 0.5; OGT_prediction (https://github.com/DavidBSauer/OGT_prediction); bbmap (https://sourceforge.net/projects/bbmap/); samtools (http://www.htslib.org/); Sibelia v.3.0.7; Circos v.0.69-8; RAxML v. 8.2.12; dRep v. 2.6.2; ARB v.6.1; clustalw v.2.1; MAFFT v7.475; iTol (webserver); trimAl v1.4.1; IQtree v2.1.12; EggNOG mapper v.2; CRISPRCasTyper v.1; PATRIC package; AlphaFold2; RoseTTAFold5 v.1.1.0; ChimeraX; vCONTACT v.2.0; CheckV v.1.0.1; Clinker v. 0.0.23; R v.4.2.1; bowtie2 v.2.4.2

For manuscripts utilizing custom algorithms or software that are central to the research but not yet described in published literature, software must be made available to editors and reviewers. We strongly encourage code deposition in a community repository (e.g. GitHub). See the Nature Portfolio guidelines for submitting code & software for further information.

Data

Policy information about availability of data

All manuscripts must include a data availability statement. This statement should provide the following information, where applicable:

- Accession codes, unique identifiers, or web links for publicly available datasets
- A description of any restrictions on data availability
- For clinical datasets or third party data, please ensure that the statement adheres to our policy

Raw metagenome reads, assembled metagenome bins and virus sequence data are available in GenBank under BioProject accession numbers PRJNA875076 and PRJNA721962. Complete ANME-1 virus genomes from Pescadero basin can be found on GenBank under accession numbers OP413838, OP413839, OP413840, OP413841, OP548099, and OP548100. CRISPR spacer sequences of ANME-1 and all genomic sequences of ANME-1 MGEs are also provided in the supplementary material. For virus genomic analysis the following databases were used in this study: Protein Data Bank (concretely the protein PM2, PDB id: 2vvf; https://www.rcsb.org/structure/2vvf), CDD v3.18, PHROG (https://phrogs.lmge.uca.fr/) and uniprot_sprot_vir70 (09/02/2021).

Human research participants

Policy information about studies involving human research participants and Sex and Gender in Research.

Reporting on sex and gender	Not applicable for this study
Population characteristics	Not applicable for this study
Recruitment	Not applicable for this study
Ethics oversight	Not applicable for this study

Note that full information on the approval of the study protocol must also be provided in the manuscript.

Field-specific reporting

Please select the one below that is the best fit for your research. If you are not sure, read the appropriate sections before making your selection.

☐ Life sciences ☐ Behavioural & social sciences ☒ Ecological, evolutionary & environmental sciences

For a reference copy of the document with all sections, see nature.com/documents/nr-reporting-summary-flat.pdf

Ecological, evolutionary & environmental sciences study design

All studies must disclose on these points even when the disclosure is negative.

Study description	Metagenomic analysis of hydrothermal vent rocks
Research sample	Sediment and Rocks collected from hydrothermal vents. The samples were chosen due to their geographical proximity to the vents with diffusive venting, which provide nutrients that fuel the local ecosystem. Some of the rocks were incubated under anoxic conditions.
Sampling strategy	Samples were collected in the field and preserved until DNA extraction in the laboratory. Sample sizes were empirically determined, typically 5 g, to allow extraction of sufficient amount of DNA. For incubated samples, sample sizes were also empirical determined, typically 1ml in volume, to allow extraction of sufficient amount of DNA while causing the least amount of disturbance to the existing microbiome.
Data collection	Metagenomic sequencing data via Illumina HiSeq4000 were collected by QuickBiology (Pasadena, CA, USA). Metagenomic sequencing data for incubated samples was conducted via Oxford Nanopore PromethION by Novogene Inc.
Timing and spatial scale	The sampling of the initial rock and sediment samples were carried out at the Auka vent field, Pescadero basin, Baja California Mexico on October 2017 and November 2018 (see Supplementary Table 1 for details).The sampling of rock incubations were sampled inside of the anaerobic chamber at Caltech between November 8, 2018 and December 15, 2019 with an increasing interval from 3 weeks to 8 months.
Data exclusions	All sequencing data were used for analyses without exclusion.
Reproducibility	The paper focuses on bioinformatics analyses, and all analyses can be reproduced using publicly available software packages provided in the Methods section. No specific incubation conditions had experimental replicates.

Randomization	The experiments were designed to discover novel organisms from any possible condition. The work does not focus on the effect of environmental parameters.
Blinding	We do not carry out randomized testing on experimental subjects, as the experiments were designed to discover novel organisms from any possible condition. There is no visual link between the samples and the microbes of interest, and there are usually 2 months between the time of sampling and the time of sequencing data output, blinding neither increase nor decrease bias.

Did the study involve field work? ☒ Yes ☐ No

Field work, collection and transport

Field conditions	Field sites are 3.6 km below sea level, collected at natural conditions on the dates and location provided in the Methods section and Supplementary Table 1.
Location	[23.956094 N 108.86192 W][23.954036 N 108.86296 W][23.942356 N 108.855825 W][23.954027 N 108.863324 W]
Access & import/export	Sample collection permits for FK181031 (25/07/2018) were granted by la Dirección General de Ordenamiento Pesquero y Acuícola, Comisión Nacional de Acuacultura y Pesca (CONAPESCA: Permiso de Pesca de Fomento No. PPFE/DGOPA-200/18) and la Dirección General de Geografía y Medio Ambiente, Instituto Nacional de Estadística y Geografía (INEGI: Autorización EG0122018), with the associated Diplomatic Note number 18-2083 (CTC/07345/18) from la Secretaría de Relaciones Exteriores - Agencia Mexicana de Cooperación Internacional para el Desarrollo / Dirección General de Cooperación Técnica y Científica. Sample collection permit for cruise NA091 (18/04/2017) was obtained by the Ocean Exploration Trust under permit number EG0072017.
Disturbance	Samples were collected outside the major chimney area to result in minimal influence on the macrofauna and the structural integrity of the chimneys.

Reporting for specific materials, systems and methods

We require information from authors about some types of materials, experimental systems and methods used in many studies. Here, indicate whether each material, system or method listed is relevant to your study. If you are not sure if a list item applies to your research, read the appropriate section before selecting a response.

Materials & experimental systems

n/a	Involved in the study	
☒	☐	Antibodies
☒	☐	Eukaryotic cell lines
☒	☐	Palaeontology and archaeology
☒	☐	Animals and other organisms
☒	☐	Clinical data
☒	☐	Dual use research of concern

Methods

n/a	Involved in the study	
☒	☐	ChIP-seq
☒	☐	Flow cytometry
☒	☐	MRI-based neuroimaging
