# Supplementary material for: Evolutionary diversification of methanotrophic ANME-1 archaea and their expansive virome
Source: Nat Microbiol. 2023 Jan 19;8(2):231–45. doi: 10.1038/s41564-022-01297-4 (PMC9894754; doi:10.1038/s41564-022-01297-4)
Supplement: Supplementary file 3 — Peer Review File [file 41564_2022_1297_MOESM3_ESM.doc]

Peer Review Information

**Journal: Nature Microbiology**

**Manuscript Title:** NMICROBIOL-22061422B

**Corresponding author name(s): Mr Rafael Laso-Pérez, Fabai Wu, Mart Krupovic, Victoria J. Orphan**

**Reviewer Comments & Decisions:**

| **Decision Letter, initial version:** | | | |
| --- | --- | --- | --- |
|  | **Message:** | 22nd August 2022  *Please ensure you delete the link to your author homepage in this e-mail if you wish to forward it to your co-authors.  Dear Mr Laso-Pérez,  Thank you for your patience while your manuscript "Evolutionary Diversification of Methanotrophic Ca. Methanophagales (ANME-1) and Their Expansive Virome" was under peer-review at Nature Microbiology. I sincerely apologize for the delay, but despite the time it has taken, I'm pleased to be able to get back to you with some good news. Your work has now been seen by 2 referees, whose expertise and comments you will find at the of this email. They both agree that your work is of interest, though they do raise some important points that should be addressed. We are very interested in the possibility of publishing your study in Nature Microbiology, but would like to consider your response to these concerns in the form of a revised manuscript before we make a final decision on publication.  In particular, you will see that Reviewer #2 has some concerns regarding the phylogenetic classifications and contamination in genomes, while Reviewer #1 felt that the portions of the manuscript discsussing ecology and evolution should be made more prominent, relative to the taxonomy of the virus. The referees’ reports are clear and the issues should be straightforward to address.  We are committed to providing a fair and constructive peer-review process. Do not hesitate to contact us if there are specific requests from the reviewers that you believe are technically impossible or unlikely to yield a meaningful outcome.  If you have not done so already please begin to revise your manuscript so that it conforms to our Article format instructions at http://www.nature.com/nmicrobiol/info/final-submission/  The usual length limit for a Nature Microbiology Article is six display items (figures or tables) and 3,000 words. We have some flexibility, and can allow a revised manuscript at 3,500 words, but please consider this a firm upper limit. There is a trade-off of ~250 words per display item, so if you need more space, you could move a Figure or Table to Supplementary Information.  Some reduction could be achieved by focusing any introductory material and moving it to the start of your opening ‘bold’ paragraph, whose function is to outline the background to your work, describe in a sentence your new observations, and explain your main conclusions. The discussion should also be limited. Methods should be described in a separate section following the discussion, we do not place a word limit on Methods.  Nature Microbiology titles should give a sense of the main new findings of a manuscript, and should not contain punctuation. Please keep in mind that we strongly discourage active verbs in titles, and that they should ideally fit within 90 characters each (including spaces).  We strongly support public availability of data. Please place the data used in your paper into a public data repository, if one exists, or alternatively, present the data as Source Data or Supplementary Information. If data can only be shared on request, please explain why in your Data Availability Statement, and also in the correspondence with your editor. For some data types, deposition in a public repository is mandatory - more information on our data deposition policies and available repositories can be found at https://www.nature.com/nature-research/editorial-policies/reporting-standards#availability-of-data.  Please include a data availability statement as a separate section after Methods but before references, under the heading "Data Availability”. This section should inform readers about the availability of the data used to support the conclusions of your study. This information includes accession codes to public repositories (data banks for protein, DNA or RNA sequences, microarray, proteomics data etc…), references to source data published alongside the paper, unique identifiers such as URLs to data repository entries, or data set DOIs, and any other statement about data availability. At a minimum, you should include the following statement: “The data that support the findings of this study are available from the corresponding author upon request”, mentioning any restrictions on availability. If DOIs are provided, we also strongly encourage including these in the Reference list (authors, title, publisher (repository name), identifier, year). For more guidance on how to write this section please see: http://www.nature.com/authors/policies/data/data-availability-statements-data-citations.pdf  To improve the accessibility of your paper to readers from other research areas, please pay particular attention to the wording of the paper’s opening bold paragraph, which serves both as an introduction and as a brief, non-technical summary in about 150 words. If, however, you require one or two extra sentences to explain your work clearly, please include them even if the paragraph is over-length as a result. The opening paragraph should not contain references. Because scientists from other sub-disciplines will be interested in your results and their implications, it is important to explain essential but specialised terms concisely. We suggest you show your summary paragraph to colleagues in other fields to uncover any problematic concepts.  If your paper is accepted for publication, we will edit your display items electronically so they conform to our house style and will reproduce clearly in print. If necessary, we will re-size figures to fit single or double column width. If your figures contain several parts, the parts should form a neat rectangle when assembled. Choosing the right electronic format at this stage will speed up the processing of your paper and give the best possible results in print. We would like the figures to be supplied as vector files - EPS, PDF, AI or postscript (PS) file formats (not raster or bitmap files), preferably generated with vector-graphics software (Adobe Illustrator for example). Please try to ensure that all figures are non-flattened and fully editable. All images should be at least 300 dpi resolution (when figures are scaled to approximately the size that they are to be printed at) and in RGB colour format. Please do not submit Jpeg or flattened TIFF files. Please see also 'Guidelines for Electronic Submission of Figures' at the end of this letter for further detail.  Figure legends must provide a brief description of the figure and the symbols used, within 350 words, including definitions of any error bars employed in the figures.  When submitting the revised version of your manuscript, please pay close attention to our href="https://www.nature.com/nature-research/editorial-policies/image-integrity">Digital Image Integrity Guidelines.</a> and to the following points below:  -- that unprocessed scans are clearly labelled and match the gels and western blots presented in figures. -- that control panels for gels and western blots are appropriately described as loading on sample processing controls -- all images in the paper are checked for duplication of panels and for splicing of gel lanes.   Finally, please ensure that you retain unprocessed data and metadata files after publication, ideally archiving data in perpetuity, as these may be requested during the peer review and production process or after publication if any issues arise.   Please include a statement before the acknowledgements naming the author to whom correspondence and requests for materials should be addressed.  Finally, we require authors to include a statement of their individual contributions to the paper -- such as experimental work, project planning, data analysis, etc. -- immediately after the acknowledgements. The statement should be short, and refer to authors by their initials. For details please see the Authorship section of our joint Editorial policies at http://www.nature.com/authors/editorial_policies/authorship.html  When revising your paper:  * include a point-by-point response to any editorial suggestions and to our referees. Please include your response to the editorial suggestions in your cover letter, and please upload your response to the referees as a separate document.  * ensure it complies with our format requirements for Letters as set out in our guide to authors at www.nature.com/nmicrobiol/info/gta/  * state in a cover note the length of the text, methods and legends; the number of references; number and estimated final size of figures and tables  * resubmit electronically if possible using the link below to access your home page:  [redacted]  Please ensure that all correspondence is marked with your Nature Microbiology reference number in the subject line.  Nature Microbiology is committed to improving transparency in authorship. As part of our efforts in this direction, we are now requesting that all authors identified as ‘corresponding author’ on published papers create and link their Open Researcher and Contributor Identifier (ORCID) with their account on the Manuscript Tracking System (MTS), prior to acceptance. This applies to primary research papers only. ORCID helps the scientific community achieve unambiguous attribution of all scholarly contributions. You can create and link your ORCID from the home page of the MTS by clicking on ‘Modify my Springer Nature account’. For more information please visit please visit <a href="http://www.springernature.com/orcid">www.springernature.com/orcid</a>.  We hope to receive your revised paper within three weeks. If you cannot send it within this time, please let us know.  We look forward to hearing from you soon.  [redacted] *******************  Reviewer Expertise:  Referee #1: marine archaea, viral metagenome analysis, hydrothermal microbial ecology  Referee #2: viral metagenomics, hydrothermal microbial ecology   Reviewers Comments:  Reviewer #1 (Remarks to the Author):  This study is a well written and very well that expands the genomic diversity of ANME archaea and their viruses. I found the taxonomy of the virus to detract from the more interesting parts like the ecology and evolution, but it’s a matter of taste I suppose. Not saying they should be named, just referring to the flow of the main text. Otherwise, I couldn’t find any major issues that I felt needed to be addressed. I think it’s important because it’s the first real examination of ANME viruses on a broad scale.  Ln 115- Was this really rRNA, or rRNA genes?    Reviewer #2 (Remarks to the Author):  In this manuscript the authors report on the analysis of Archaeal genomes from the order Methanophagales as well as their associated virome, both of which represent novel biological diversity from poorly characterized ecosystems. The data is relevant, the analysis were properly designed, and the results support the conclusions. Furthermore, the paper is clear, concise, and very well-written. Nevertheless, some issues related to the interpretation and validity of some results must be properly addressed to make it suitable for publication.   Figure 1: Tree topology suggests that some species (e.g. 5, 18) are not monophyletic. Please explain the rationale for assigning these genomes to the same species if this claim is not backed up by the phylogeny. Also in Figure 1 it would be better to have the species assignment at the leaf ids, for all leaves, and not on the far right side of the plot.  Table S1: The QC data of the genomes suggests that there might have been issues when selecting which genomes to use for phylogenomic analysis. Specifically, why include genomes with high contamination (i.e. > 5%) in the analysis? This has the potential to drastically affect the phylogenomic reconstruction, rendering the results not valid.  Ln 103-105: If the ANME subclades share a common and exclusive ancestor, thus making them a monophyletic group, how can the authors claim that ANME-1c is more closely related to the sister clades ‘Ca. Alkanophagales’ and ‘Ca. Syntrophoarchaeales’ than ANME-1a and ANME-1b?  Ln 277-280: Do these new viral families, and the parent clades proposed, meet the necessary criteria to be defined as new taxa according to the ICTV?  Ln 399-400: This is the first time in the text that the potential for autotrophic metabolism in ANME-1 is mentioned. It should be mentioned in the introduction before discussing the potential role of the viral metabolic genes in this section.  Ln 452: Do these organisms have both the Calvin-Benson-Basham and Wood-Ljungdahl pathways?  Ln 576: Are the species clustering obtained with dRep consistent with the GTDBtk classification?  Ln 611-634: In this section the authors describe the use of CRISPR spacers to identify mobile genetic elements, which should include viruses, plasmids, and others. But there is no information regarding how viruses were separated from other MGE. As it is written, it appears all were used for subsequent analysis in the “Virus protein annotations”, “Mobilome network analysis and evaluation”, and “Virus genome alignment” sections |  |

**Author Rebuttal to Initial comments**

# The reviewer’s comments are in blue and our response in black Reviewer #1 (Remarks to the Author):

This study is a well written and very well that expands the genomic diversity of ANME archaea and their viruses. I found the taxonomy of the virus to detract from the more interesting parts like the ecology and evolution, but it’s a matter of taste I suppose. Not saying they should be named, just referring to the flow of the main text. Otherwise, I couldn’t find any major issues that I felt needed to be addressed. I think it’s important because it’s the first real examination of ANME viruses on a broad scale.

We are grateful for the kind words from the reviewer. Following the advice above, we have moved almost all detailed reasoning for viral taxonomic classification and descriptions of viruses into the methods section and the supplementary text. We thank the reviewer for the advice and do agree that this now makes the main text more streamlined, and that the ecology and evolution aspects of both ANME archaea and viruses more prominently highlighted.

Ln 115‐ Was this really rRNA, or rRNA genes?

Indeed the surveys refers to the rRNA gene and not to rRNA. We have made modifications in the text accordingly. This now correspond to line 87.

# Reviewer #2 (Remarks to the Author):

In this manuscript the authors report on the analysis of Archaeal genomes from the order Methanophagales as well as their associated virome, both of which represent novel biological diversity from poorly characterized ecosystems. The data is relevant, the analysis were properly designed, and the results support the conclusions. Furthermore, the paper is clear, concise, and very well‐written. Nevertheless, some issues related to the interpretation and validity of some results must be properly addressed to make it suitable for publication.

We thank the reviewer for the positive comments on the manuscript.

Figure 1: Tree topology suggests that some species (e.g. 5, 18) are not monophyletic. Please explain the rationale for assigning these genomes to the same species if this claim is not backed up by the phylogeny. Also in Figure 1 it would be better to have the species assignment at the leaf ids, for all leaves, and not on the far right side of the plot.

Indeed the tree topology suggest that some species appears to not be monophyletic in the previous figure. Overall, species boundaries are defined based on average nucleotide identity (ANI), which is what was done in this study, and the tree topology between related species can somewhat depend on the choice of reference strain and the amount of markers available in these genomes. Considering that species‐level diversity does not contribute to our conclusions, except for the case of ANME‐1c, we decided to remove the species definitions for ANME‐1a and ANME‐1b from the manuscript and work at the genus level (for instance to obtain the growth temperature of the different groups, figure S2; or genome abundances, Figure 2) avoiding any discordance between the ANI species definition and the tree topology. We now only indicate the species number for those of ANME‐1c and at the leaves facilitating the readability of the figure.

Table S1: The QC data of the genomes suggests that there might have been issues when selecting which genomes to use for phylogenomic analysis. Specifically, why include genomes with high contamination

(i.e. > 5%) in the analysis? This has the potential to drastically affect the phylogenomic	reconstruction, rendering the results not valid.

Indeed we have included some genomes with higher contamination scores as evaluated by CheckM, and they were inherited from a recent paper that carried our substantial analyses across ANMEs (Chadwick did in his 2022 study in Plos Biology). We did so for the convenience of a direct comparison with this paper. We have now included a new column in the Table S2 indicating the strain heterogeneity of each bin, from which we observed that the bins with high contamination usually encompass sequences from more

than one strain of the same species. The phylogenomic method that we use only chooses the marker gene with the best score hits in case several hits are detected in the bin, but we admit that genomes suspected of contamination by CheckM still could affect the phylogenomic reconstruction. Therefore, we have recalculated the phylogenomic tree, where we excluded bins with a CheckM contamination score higher than 5% or with a CheckM completeness score lower than 60% (see figure above). The topology of this new tree with selected MAGs maintains the topology of the old one.

Ln 103‐105: If the ANME subclades share a common and exclusive ancestor, thus making them a monophyletic group, how can the authors claim that ANME‐1c is more closely related to the sister clades ‘Ca. Alkanophagales’ and ‘Ca. Syntrophoarchaeales’ than ANME‐1a and ANME‐1b?

We agree that the previous sentence might not reflect the idea we wanted to express. Our results showed that ANME‐1c occupies a basal position in the ANME‐1 order. Therefore, they are the closest ANME‐1 group to the sister clades based on phylogenetic distance. Indeed all ANME‐1 groups share a common ancestor, but our phylogenomic tree seems to indicate that ANME‐1c emerged first, while ANME‐1a and ANME‐1b appeared later from an ANME‐1ab ancestor. Alternatively, ANME‐1c could have evolved slower, and thus inherited more ancestral features in common with the short‐chain‐alkane oxidizers. As described in the paper, this is not only reflected by the phylogenetic markers, but also various other physiological characteristics. Accordingly, we modified the sentence to the following (Line 76‐79): *“We name this clade*

*´Candidatus Methanoxibalbaceae´, or ANME‐1c, which given its basal position, is the phylogenetically closest ANME‐1 to the sister orders of non‐methane alkane degraders Alkanophagales and Syntrophoarchaeales”*

Ln 277‐280: Do these new viral families, and the parent clades proposed, meet the necessary criteria to be defined as new taxa according to the ICTV?

A formal taxa classification to be approved by ICTV requires complete virus genomes. To this end, we have such representatives for 5 new families across all 4 viral morphotypes, while the remaining 11 families only have incomplete (or unconfirmed due to lack of terminal repeats) and thus are named candidate families. The 5 new families are highlighted in Fig. 4c, and their taxonomic classifications which we have formally proposed to the ICTV in July 2022 are detailed in the supplementary table 12 and in the section ‘Taxonomic description of proposed ANME‐1 virus orders and families with representative complete genomes’. The proposed taxa have been approved by the Executive Committee of the ICTV and are awaiting ratification by the full ICTV membership.

Ln 399‐400: This is the first time in the text that the potential for autotrophic metabolism in ANME‐1 is mentioned. It should be mentioned in the introduction before discussing the potential role of the viral metabolic genes in this section.

We thank the reviewer for spotting this potential line of interest. CbbQ‐like proteins we mention in the text belong to the broader NirQ/NorQ/CbbQ chaperones; NirQ involved in denitrification was shown previously to be able to activate Rubisco when ectopically expressed. However, given that Rubisco was not found in ANME‐1, we decided to omit such a mention in the text to avoid extended speculations. Along with the many viral features, this protein was the only functional feature from nonviral MGEs mentioned in the paper. We thus decide to omit the mention of this gene in the present paper, and leave all nonviral MGEs for future studies.

Additionally, to comply with the length limit set by the journal, we have moved the detailed descriptions of all other viral AMGs to the supplementary text, except for ThyX.

Ln 452: Do these organisms have both the Calvin‐Benson‐Basham and Wood‐Ljungdahl pathways?

No, to our knowledge ANME‐1 have the Wood‐Ljungdahl pathway and lack the key rubisco enzyme from the CBB cycle.

Ln 576: Are the species clustering obtained with dRep consistent with the GTDBtk classification?

Most of the ANME‐1 MAGs do not reach the level of species when we classified them with the GTDB‐tk. For those few that reach the species level, there are cases in which dRep is consistent with GTDBtk (old species 3 – MAGs 11719B_bin4_ANME1, ANME‐1 ex4572‐4 and ANME‐1 G37), and others in which this is not the case. For instance, dRep describes species 5 (ANME‐1 WYZ‐LMO12, ANME‐1 WYZ‐LMO13), 6 (ANME‐1 B64_G16, FWG148_ANME1) and 7 (ANME‐1 M5.MMPM), while for GTDB‐tk all these MAGs belong to the same species (s QEXZ01 sp003601795) with the exception of ANME‐1 WYZ‐LMO12 that only belongs to the same genus (g QEXZ01). As discussed in the comments above related to Figure 1 topology, there can be various factors for the minor differences in species classification for MAGs, and GTDB‐tk also continuously update their reference species. Given that the exact division of species above

do not influence our conclusions, we now use the genus level definition (except for the case of ANME‐1c) as indicated in Supplementary Table 2.

Ln 611‐634: In this section the authors describe the use of CRISPR spacers to identify mobile genetic elements, which should include viruses, plasmids, and others. But there is no information regarding how viruses were separated from other MGE. As it is written, it appears all were used for subsequent analysis in the “Virus protein annotations”, “Mobilome network analysis and evaluation”, and “Virus genome alignment” sections.

We thank the reviewer for pointing this out. Indeed all preliminary analyses for the network analyses and genome annotations were done on all MGEs. The viruses were then separated from the other MGEs through the identification of viral structural proteins. It is now specified in the main text line xxx and we modified the methods sections to make this more explicit. The headers are now correspondingly ‘MGE network analysis and evaluation’, ‘MGE annotation and virus identification’ and ‘Genome‐scale virus comparisons’.

**Decision Letter, first revision:**

| **Message:** | Our ref: NMICROBIOL-22061422A  11th October 2022  Dear Dr. Laso-Pérez,  Thank you for your patience as we’ve prepared the guidelines for final submission of your Nature Microbiology manuscript, "Evolutionary Diversification of Methanotrophic Ca. Methanophagales (ANME-1) and Their Expansive Virome" (NMICROBIOL-22061422A). Please carefully follow the step-by-step instructions provided in the attached file, and add a response in each row of the table to indicate the changes that you have made. Please also check and comment on any additional marked-up edits we have proposed within the text. Ensuring that each point is addressed will help to ensure that your revised manuscript can be swiftly handed over to our production team.  We would like to start working on your revised paper, with all of the requested files and forms, as soon as possible (preferably within two weeks). Please get in contact with us if you anticipate delays.  When you upload your final materials, please include a point-by-point response to any remaining reviewer comments.  If you have not done so already, please alert us to any related manuscripts from your group that are under consideration or in press at other journals, or are being written up for submission to other journals (see: https://www.nature.com/nature-research/editorial-policies/plagiarism#policy-on-duplicate-publication for details).  In recognition of the time and expertise our reviewers provide to Nature Microbiology’s editorial process, we would like to formally acknowledge their contribution to the external peer review of your manuscript entitled "Evolutionary Diversification of Methanotrophic Ca. Methanophagales (ANME-1) and Their Expansive Virome". For those reviewers who give their assent, we will be publishing their names alongside the published article.  Nature Microbiology offers a Transparent Peer Review option for new original research manuscripts submitted after December 1st, 2019. As part of this initiative, we encourage our authors to support increased transparency into the peer review process by agreeing to have the reviewer comments, author rebuttal letters, and editorial decision letters published as a Supplementary item. When you submit your final files please clearly state in your cover letter whether or not you would like to participate in this initiative. Please note that failure to state your preference will result in delays in accepting your manuscript for publication.  **Cover suggestions**  As you prepare your final files we encourage you to consider whether you have any images or illustrations that may be appropriate for use on the cover of Nature Microbiology.  Covers should be both aesthetically appealing and scientifically relevant, and should be supplied at the best quality available. Due to the prominence of these images, we do not generally select images featuring faces, children, text, graphs, schematic drawings, or collages on our covers.  We accept TIFF, JPEG, PNG or PSD file formats (a layered PSD file would be ideal), and the image should be at least 300ppi resolution (preferably 600-1200 ppi), in CMYK colour mode.  If your image is selected, we may also use it on the journal website as a banner image, and may need to make artistic alterations to fit our journal style.  Please submit your suggestions, clearly labeled, along with your final files. We’ll be in touch if more information is needed.   Nature Microbiology has now transitioned to a unified Rights Collection system which will allow our Author Services team to quickly and easily collect the rights and permissions required to publish your work. Approximately 10 days after your paper is formally accepted, you will receive an email in providing you with a link to complete the grant of rights. If your paper is eligible for Open Access, our Author Services team will also be in touch regarding any additional information that may be required to arrange payment for your article.  Please note that you will not receive your proofs until the publishing agreement has been received through our system.  Please note that <i>Nature Microbiology</i> is a Transformative Journal (TJ). Authors may publish their research with us through the traditional subscription access route or make their paper immediately open access through payment of an article-processing charge (APC). Authors will not be required to make a final decision about access to their article until it has been accepted. <a href="https://www.springernature.com/gp/open-research/transformative-journals"> Find out more about Transformative Journals</a>  **Authors may need to take specific actions to achieve <a href="https://www.springernature.com/gp/open-research/funding/policy-compliance-faqs"> compliance</a> with funder and institutional open access mandates.** If your research is supported by a funder that requires immediate open access (e.g. according to <a href="https://www.springernature.com/gp/open-research/plan-s-compliance">Plan S principles</a>) then you should select the gold OA route, and we will direct you to the compliant route where possible. For authors selecting the subscription publication route, the journal’s standard licensing terms will need to be accepted, including <a href="https://www.nature.com/nature-portfolio/editorial-policies/self-archiving-and-license-to-publish">self-archiving policies</a>. Those licensing terms will supersede any other terms that the author or any third party may assert apply to any version of the manuscript.   For information regarding our different publishing models please see our <a href="https://www.springernature.com/gp/open-research/transformative-journals"> Transformative Journals </a> page. If you have any questions about costs, Open Access requirements, or our legal forms, please contact ASJournals@springernature.com.  [redacted]  If you have any further questions, please feel free to contact me.   [redacted]       Reviewer #1: Remarks to the Author: All of my comments have been address, and I think that moving the taxonomy to the Suppl improves paper. I strongly feel this is worthy of publication in NM.    Reviewer #2: Remarks to the Author: All the topics raised in my initial review wer eproperly addressed. I endorse this version for publication. |
| --- | --- |

**Final Decision Letter:**

| **Message:** | 29th November 2022  Dear Dr Laso-Pérez,  I am pleased to accept your Article "Evolutionary diversification of methanotrophic ANME-1 archaea and their expansive virome" for publication in Nature Microbiology. Thank you for having chosen to submit your work to us and many congratulations.  Over the next few weeks, your paper will be copyedited to ensure that it conforms to Nature Microbiology style. We look particularly carefully at the titles of all papers to ensure that they are relatively brief and understandable.  Once your paper is typeset, you will receive an email with a link to choose the appropriate publishing options for your paper and our Author Services team will be in touch regarding any additional information that may be required. Once your paper has been scheduled for online publication, the Nature press office will be in touch to confirm the details.  After the grant of rights is completed, you will receive a link to your electronic proof via email with a request to make any corrections within 48 hours. If, when you receive your proof, you cannot meet this deadline, please inform us at rjsproduction@springernature.com immediately. You will not receive your proofs until the publishing agreement has been received through our system  Due to the importance of these deadlines, we ask you please us know now whether you will be difficult to contact over the next month. If this is the case, we ask you provide us with the contact information (email, phone and fax) of someone who will be able to check the proofs on your behalf, and who will be available to address any last-minute problems.  Acceptance of your manuscript is conditional on all authors' agreement with our publication policies (see https://www.nature.com/nmicrobiol/editorial-policies). In particular your manuscript must not be published elsewhere and there must be no announcement of the work to any media outlet until the publication date (the day on which it is uploaded onto our website).  Please note that <i>Nature Microbiology</i> is a Transformative Journal (TJ). Authors may publish their research with us through the traditional subscription access route or make their paper immediately open access through payment of an article-processing charge (APC). Authors will not be required to make a final decision about access to their article until it has been accepted. <a href="https://www.springernature.com/gp/open-research/transformative-journals"> Find out more about Transformative Journals</a>  **Authors may need to take specific actions to achieve <a href="https://www.springernature.com/gp/open-research/funding/policy-compliance-faqs"> compliance</a> with funder and institutional open access mandates.** If your research is supported by a funder that requires immediate open access (e.g. according to <a href="https://www.springernature.com/gp/open-research/plan-s-compliance">Plan S principles</a>) then you should select the gold OA route, and we will direct you to the compliant route where possible. For authors selecting the subscription publication route, the journal’s standard licensing terms will need to be accepted, including <a href="https://www.nature.com/nature-portfolio/editorial-policies/self-archiving-and-license-to-publish">self-archiving policies</a>. Those licensing terms will supersede any other terms that the author or any third party may assert apply to any version of the manuscript.   If you have any questions about our publishing options, costs, Open Access requirements, or our legal forms, please contact ASJournals@springernature.com  An online order form for reprints of your paper is available at <a href="https://www.nature.com/reprints/author-reprints.html">https://www.nature.com/reprints/author-reprints.html</a>. All co-authors, authors' institutions and authors' funding agencies can order reprints using the form appropriate to their geographical region.  We welcome the submission of potential cover material (including a short caption of around 40 words) related to your manuscript; suggestions should be sent to Nature Microbiology as electronic files (the image should be 300 dpi at 210 x 297 mm in either TIFF or JPEG format). Please note that such pictures should be selected more for their aesthetic appeal than for their scientific content, and that colour images work better than black and white or grayscale images. Please do not try to design a cover with the Nature Microbiology logo etc., and please do not submit composites of images related to your work. I am sure you will understand that we cannot make any promise as to whether any of your suggestions might be selected for the cover of the journal.  You can now use a single sign-on for all your accounts, view the status of all your manuscript submissions and reviews, access usage statistics for your published articles and download a record of your refereeing activity for the Nature journals.  To assist our authors in disseminating their research to the broader community, our SharedIt initiative provides you with a unique shareable link that will allow anyone (with or without a subscription) to read the published article. Recipients of the link with a subscription will also be able to download and print the PDF.   As soon as your article is published, you will receive an automated email with your shareable link.   With kind regards,  [redacted] |
| --- | --- |
